# Up‐regulation of lipid biosynthesis increases the oil content in leaves of *Sorghum bicolor*

**DOI:** 10.1111/pbi.12959

**Published:** 2018-07-13

**Authors:** Thomas Vanhercke, Srinivas Belide, Matthew C. Taylor, Anna El Tahchy, Shoko Okada, Vivien Rolland, Qing Liu, Madeline Mitchell, Pushkar Shrestha, Ingrid Venables, Lina Ma, Cheryl Blundell, Anu Mathew, Lisa Ziolkowski, Nathalie Niesner, Dawar Hussain, Bei Dong, Guoquan Liu, Ian D. Godwin, Jiwon Lee, Melanie Rug, Xue‐Rong Zhou, Surinder P. Singh, James R. Petrie

**Affiliations:** ^1^ CSIRO Agriculture and Food Canberra ACT Australia; ^2^ CSIRO Land and Water Canberra ACT Australia; ^3^ School of Agriculture and Food Sciences University of Queensland Brisbane QLD Australia; ^4^ Centre for Advanced Microscopy Australian National University Canberra ACT Australia

**Keywords:** triacylglycerol, *Sorghum bicolor*, leaf, WRI1, DGAT, Oleosin

## Abstract

Synthesis and accumulation of the storage lipid triacylglycerol in vegetative plant tissues has emerged as a promising strategy to meet the world's future need for vegetable oil. Sorghum (*Sorghum bicolor*) is a particularly attractive target crop given its high biomass, drought resistance and C_4_ photosynthesis. While oilseed‐like triacylglycerol levels have been engineered in the C_3_ model plant tobacco, progress in C_4_ monocot crops has been lagging behind. In this study, we report the accumulation of triacylglycerol in sorghum leaf tissues to levels between 3 and 8.4% on a dry weight basis depending on leaf and plant developmental stage. This was achieved by the combined overexpression of genes encoding the *Zea mays* WRI1 transcription factor, *Umbelopsis ramanniana* UrDGAT2a acyltransferase and *Sesamum indicum* Oleosin‐L oil body protein. Increased oil content was visible as lipid droplets, primarily in the leaf mesophyll cells. A comparison between a constitutive and mesophyll‐specific promoter driving *WRI1* expression revealed distinct changes in the overall leaf lipidome as well as transitory starch and soluble sugar levels. Metabolome profiling uncovered changes in the abundance of various amino acids and dicarboxylic acids. The results presented here are a first step forward towards the development of sorghum as a dedicated biomass oil crop and provide a basis for further combinatorial metabolic engineering.

## Introduction

Plant‐derived oils are one of the fastest growing agricultural commodities (OECD/FAO, 2017). World production of plant oils currently amounts to nearly 190 Mt with the vast majority (86%) being used for food and feed applications. The remaining 14% (26.8 Mt) serves as feedstocks for the production of biofuels and oleochemicals in an attempt to reduce our reliance on dwindling crude oil supplies. By 2026, global plant oil demand for food use is expected to increase up to 219.8 Mt which equates to a 16% increase (OECD/FAO, 2017). In the past, plant oil production has increased steadily by dedicating more agricultural acreage to oil crops. However, limitations on available arable land and growing deforestation concerns pose a challenge when trying to match future demand using current established oil crops. This has triggered a search for alternative plant oil production systems that could potentially achieve yields similar to or even higher than current oil crops.

More than 60% of the current plant oil production is sourced from triacylglycerol (TAG)‐rich seeds (OECD/FAO, 2017). The metabolic pathways leading from sugars to TAG are complex and tightly intertwined with membrane biogenesis (Li‐Beisson *et al*., 2013). Over the past decades, the majority of enzymatic steps involved in the synthesis of fatty acids, membrane and storage lipids have been characterized in the oilseed model *Arabidopsis thaliana*, while knowledge of the underlying regulatory control mechanisms and lipid fluxes is broadening (Bates, 2016; Manan *et al*., 2017; Troncoso‐Ponce *et al*., 2016). Lipid metabolism in most vegetative tissues is predominantly directed towards phospholipid biosynthesis required to maintain the function of chloroplast membrane systems that are critical for efficient photosynthesis. Unlike oilseeds, leaves accumulate low levels of storage lipids such as TAG which typically accounts for less than 1% of the total leaf lipids (Yang and Ohlrogge, 2009). Studies in *A. thaliana* leaves suggest that TAG fulfils a role as a transient metabolic pool for excess lipotoxic free fatty acids that can occur as a result of membrane turnover in response to various stress factors and tissue ageing (Fan *et al*., 2013a, 2014, 2017).

Because nonseed tissues harbour a functional TAG biosynthesis pathway and represent the majority of the above‐ground plant biomass, engineering increased levels of TAG in vegetative organs has attracted increasing attention as a novel plant oil production system (Ohlrogge and Chapman, 2011). A growing number of transgenic metabolic engineering studies have demonstrated the feasibility of increasing the oil content in various nonseed tissues (Weselake, 2016; Xu and Shanklin, 2016). Amongst these reports, multigene strategies are now clearly leading the way, culminating in the engineering of oilseed‐like TAG levels in leaves of tobacco (Vanhercke *et al*., 2017). This was achieved by the simultaneous overexpression of *WRINKLED1* (*WRI1*), a positive regulator of fatty acid synthesis (‘Push’), acyl CoA:diacylglycerol acyltransferase 1 (*DGAT1)* which catalyses the final TAG assembly step (‘Pull’), and *Oleosin‐L* oil droplet protein (‘Package’) while reducing lipid turnover by overexpression of the *LEAFY COTYLEDON2* (*LEC2*) embryogenic transcription factor or silencing of the *SUGAR DEPENDENT1* (*SDP1*) lipase (‘Protect’). Contrary to the results in *A. thaliana* and tobacco, achievements in other species thus far remain limited to small increases in TAG or total lipids contents. Examples include ryegrass (Beechey‐Gradwell, 2016; Winichayakul *et al*., 2008), corn (Alameldin *et al*., 2017), potato (Hofvander *et al*., 2016; Klaus *et al*., 2004; Liu *et al*., 2017), *Jatropha curcas* (Maravi *et al*., 2016) and rice (Singh *et al*., 2016).

High biomass C_4_ crop species such as sorghum (*Sorghum bicolor*), sugarcane (*Saccharum* spp.), *Miscanthus giganteus* and switchgrass (*Panicum virgatum*) provide particularly attractive targets for the synthesis and accumulation of oil in vegetative plant parts (Ohlrogge and Chapman, 2011). Assuming 20 ton dry biomass per hectare, the engineering of >15% oil in leaves and stems on a dry weight basis could potentially rival or surpass oil palm in terms of oil yield. The overexpression of *WRI1*,* DGAT1* and *Oleosin* transgenes while silencing two additional genes involved in starch biosynthesis pathway (*AGPase*) and lipid turnover (*PXA1*) only yielded around 2% TAG on a dry weight (DW) basis in leaves and stems of sugarcane (Zale *et al*., 2016). This outcome illustrates the need for further pathway optimization to address potential additional lipid flux bottlenecks that occur in this species.

Sorghum is the fifth most important cereal crop in the world. Grain sorghum varieties provide an important staple food and feed supply, while sweet and forage sorghum varieties are cultivated for biofuel and fodder markets, respectively (Calvino and Messing, 2012; Mathur *et al*., 2017; Smith and Frederiksen, 2000). Compared to sugarcane, sorghum is adapted to both tropical and temperate growing regions due to its higher water use efficiency and the ability to enter growth arrest when facing drought conditions. In addition, sorghum has relatively low input requirements, can grow on a wide variety of soils that are suboptimal for other crops and has a relatively short growing period of 4–5 months. Amongst potential C_4_ biofuel crops, sorghum has one of the highest photosynthetic energy conversion efficiencies (Slattery and Ort, 2015). High biomass sorghum hybrids exhibit delayed flowering during the growing season due to photoperiod sensitivity for short day lengths. The prolonged vegetative growth phase leads to biomass yields from 15 up to 60 tons DW per hectare, making sorghum an excellent target crop to engineer increased TAG levels in leaves and stems (Olson *et al*., 2012). As a first step towards developing sweet or forage sorghum as a dedicated biomass oil crop, we explored the possibility of increasing the TAG content in leaves of grain sorghum (Tx430). We chose this inbred line as a C_4_ model given its shorter life cycle, ease of backcrossing into appropriate biomass or forage sorghum cultivars and the high‐efficiency transformation protocol that was recently established in our group (Belide *et al*., 2017). Here, we report how overexpression of *WRI1*,* DGAT1* and *Oleosin‐L* results in the synthesis and accumulation of more than 8% TAG (DW) in leaves of primary transformants, visible as oil droplets primarily within mesophyll cells.

## Results

### Overexpression of WRI1, DGAT2 and Oleosin‐L in sorghum

**Figure 1:**
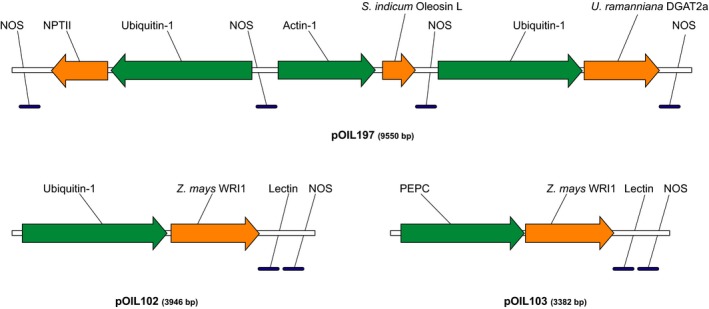
Schematic representation of the minimal expression cassettes containing *Zea mays WRI1*,* Umbelopsis ramanniana DGAT2a* and *Sesamum indicum Oleosin‐L*. Orange arrows represent transgenes, promoter regions are shown as green arrows and blue lines indicate terminator sequences. Ubiquitin‐1, *Z. mays* Ubiquitin‐1 promoter; Actin‐1, *O. sativa* Actin‐1 promoter; PEPC, *Z. mays* PEPC promoter; Nos, *Rhizobium radiobacter* Nopaline synthase polyadenylation signal; Lectin, *Glycine max* Lectin polyadenylation signal.

We designed a series of expression constructs for rapid and modular testing of different gene and promoter combinations in the primary generation (T_0_) of *S. bicolor* Tx430 by biolistic co‐transformation. We describe here the results obtained with three of these genetic constructs. The first vector, pOIL197, contained the *U. ramanniana* DGAT2a and *S. indicum* Oleosin‐L expression cassettes in addition to the NPTII selectable marker (Figure 1). Two other vectors, pOIL102 and pOIL103, contained the *Z. mays WRI1* gene under the control of the *Z. mays* Ubiquitin‐1 or PEPC promoter, respectively. The PEPC promoter was chosen to restrict *WRI1* expression under light conditions to the mesophyll cells where initial carbon fixation by the PEP carboxylase (PEPC) occurs in C_4_ plants. On the other hand, the constitutive ubiquitin‐1 promoter is expected to result in strong expression in both mesophyll and bundle sheath cells.

A total of 9, 37, 47 and 205 T_0_ plants were obtained after transformation of *S. bicolor* with pOIL102 (‘01’ lines), pOIL102+pOIL197 (‘02’ lines), pOIL103+pOIL197 (‘03’ lines) and pOIL197 (‘197’ lines), respectively. The presence or absence of all three transgenes was determined by ddPCR analysis (Table S2). Of note, we observed up to 30% mortality rate at rooting stage during tissue culture following transformation with pOIL103+pOIL197 due to unknown reasons.

### Increased lipid content in transgenic sorghum leaves

**Figure 2:**
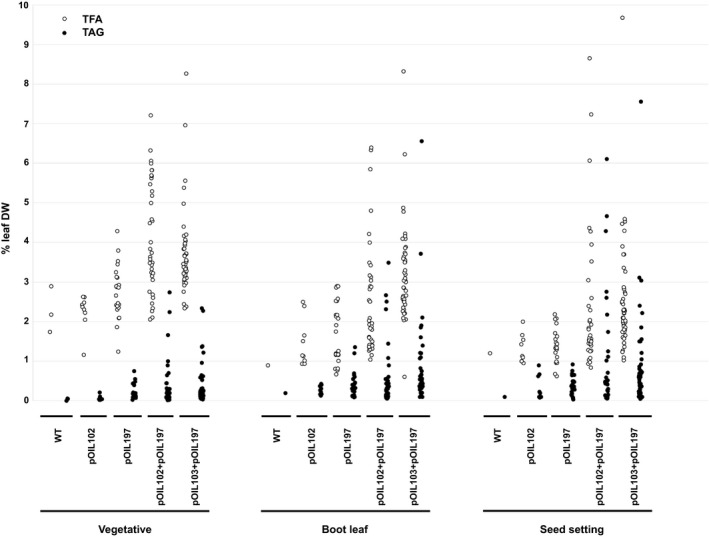
Levels of total lipids (TFA) and triacylglycerol (TAG) on a dry weight basis (DW) in leaves of *Sorghum bicolor* primary transformants at three stages during development. Sorghum was transformed with pOIL102, pOIL197, pOIL102+pOIL197 or pOIL103+pOIL197. ‘Vegetative’, ‘Boot leaf’ and ‘Seed setting’ corresponded to stages 3–4, 5 and 7–8 of Vanderlip and Reeves (1972). Data are based on single leaf measurements for each transgenic line.

Transgenic plants were grown in the glasshouse, and the lowest fully green leaves were sampled at three different stages during development: stage 3–4 (‘vegetative’), stage 5 (‘boot leaf’) and stage 8 (‘seed setting’) (Vanderlip and Reeves, 1972). Initial screening revealed many lines with elevated lipid contents in leaf tissues (Figure 2). While wild‐type controls and negative transformants, as identified by ddPCR, displayed TAG levels below 0.2% (leaf DW) throughout the entire life cycle, maximum TAG levels amongst pOIL102+pOIL197 and pOIL103+pOIL197 populations reached 6.1% (line 02–10) and 7.6% (line 03–31) (leaf DW) at seed setting stage, respectively. Corresponding TFA levels in these transgenic events equalled 8.7 and 9.7% compared to a maximum of 1.6% TFA (leaf DW) in leaves of negative controls. Unlike previous results obtained in tobacco (Vanhercke *et al*., 2014), increases in TAG levels throughout plant development were less pronounced. Total lipids in many transgenic lines gradually decreased during the life cycle. Interestingly, transformation with either pOIL102 or pOIL197 only resulted in a small increase in TFA and TAG contents which decreased as plants progressed to boot leaf and seed setting stages.

Due to the use of biolistic transformation as a rapid transgene evaluation system, not surprisingly, many transgenic sorghum plants contained high transgene copy numbers, as determined by ddPCR (Table S2). In addition, we observed varying degrees of sterility amongst the transgenic lines, possibly as a result of multiple transgene insertions. We therefore performed a detailed analysis on vegetatively propagated tillers obtained from selected primary transformants, similarly to earlier work in sugarcane and potato (Liu *et al*., 2017; Zale *et al*., 2016). To this end, tillers were propagated allowing for triplicate analyses of TAG and TFA levels. Furthermore, for the remainder of the study, we predominantly focussed on the boot leaf stage as this is a distinct and easily identifiable time point during development that allows for rigorous comparison between the different transgenic lines.

Quantification of the lipid content in triplicate leaves from established tillers confirmed elevated TAG and TFA contents in several independent lines co‐transformed with either pOIL102+pOIL197 or pOIL103+pOIL197. The highest levels were observed in line 03–31 confirming earlier screening results. Leaves of this line contained on average 6.9% TFA and 4.6% TAG (leaf DW) at boot leaf stage (Figure S1). This corresponded to an 89.4‐fold increase in TAG content compared to wild‐type control leaves at the same developmental stage. Sorghum plants transformed either with pOIL197 or pOIL102 accumulated up to 0.5% TAG and 3.5% TFA (leaf DW).

### Transgene expression and correlation with lipid content

Transgene expression levels were determined in propagated tillers of selected lines by ddPCR (Table S3). In the majority of transgenic lines, *DGAT2a* was typically expressed at a higher level than the *WRI1* transgene. *Oleosin‐L* expression was either low or absent. Total lipid and TAG contents at boot leaf stage were used to calculate correlation coefficients (Table 1). Both *WRI1* and *DGAT2a* expression showed a significant positive correlation (≥0.79) with TAG levels amongst pOIL102+pOIL197 and pOIL103+pOIL197 transgenic populations. A significant albeit slightly weaker correlation (≥0.57) was observed for TFA content and *WRI1* or *DGAT2a* expression. *Oleosin‐L* expression was low in most transgenic lines.

**Table 1:** Pearson correlation coefficients† for transgene expression and total lipids (TFA) or triacylglycerol (TAG) content (% leaf dry weight) at boot leaf stage for re‐established tillers

Constructs	Lipid class	WRI1	DGAT2	Oleosin‐L
pOIL102+pOIL197‡	TFA	0.7737** (0.000269)	0.6019* (0.010576)	0.0772 (0.768377)
	TAG	0.8252** (0.000045)	0.7903** (0.00016)	0.3689 (0.145078)
pOIL103+pOIL197‡	TFA	0.5675* (0.034286)	0.6764** (0.007902)	−0.2299 (0.430987)
	TAG	0.8018** (0.000563)	0.9428** (< 0.00001)	−0.0154 (0.95941)

*P < 0.05; **P < 0.01.

^†^Significance levels are indicated between brackets.

^‡^ *n* = 17 (pOIL102+pOIL197) or 14 (pOIL103+pOIL197).

### Sorghum leaf lipidomic changes

**Figure 3:**
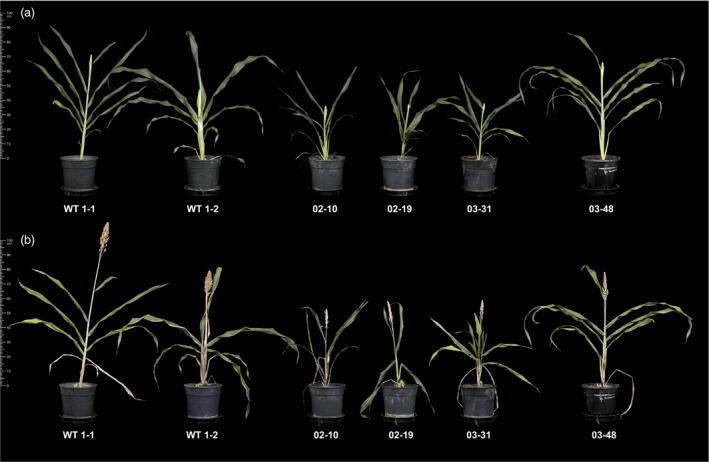
Propagated tillers from wild‐type and four independent transgenic *Sorghum bicolor* lines at boot leaf (a) and seed setting (b) stages. Transgenic ‘02’ and ‘03’ events were transformed with pOIL102+pOIL197 or pOIL103+pOIL197, respectively. Scale bar: 1 m.

We next established replicate tillers of four selected transgenic lines (02–10, 02–19, 03–31 and 03–48) exhibiting varying elevated TAG levels for a more detailed lipid analysis, including an investigation of possible changes occurring in the wider leaf lipidome. Propagated tillers of transgenic primary transformants were smaller compared to tillers obtained from wild‐type controls plants with the exception of line 03–48 (Figure 3). Quantification by GC‐FID of TAG and TFA levels in triplicate leaves confirmed increases in both lipid fractions (Figures S2 and S3). Maximum average TAG levels in triplicate leaves (% DW) of lines 02–19 and 03–31 sampled at boot leaf stage were 2.8% and 5.2%, respectively. In all transgenic lines, linoleic acid (C18:2^Δ9,12^) was increased at the expense of α‐linolenic acid (C18:3^Δ9,12,15^). However, we detected distinct differences in the levels of two other major fatty acids, palmitic acid (C16:0) and oleic acid (C18:1^Δ9^). Lines 02–10 and 02–19 contained increased proportions of oleic acid, while palmitic acid was typically elevated in the TFA and TAG fractions of 03–31 and 03–48 leaves. Lipid quantification in leaf and stem tissues at seed setting stage revealed considerable leaf‐to‐leaf variation (Figure S4). Lower TFA and TAG contents were observed in older leaves of wild‐type and transgenic propagated tillers. The highest TFA and TAG levels were detected in the flag leaf of line 03–31 at seed setting equalling 9.9% and 8.4% on a DW basis, respectively. Transgenic stem tissues contained less than 3% total lipids (DW) compared to 0.3% in wild‐type stems.

**Figure 4:**
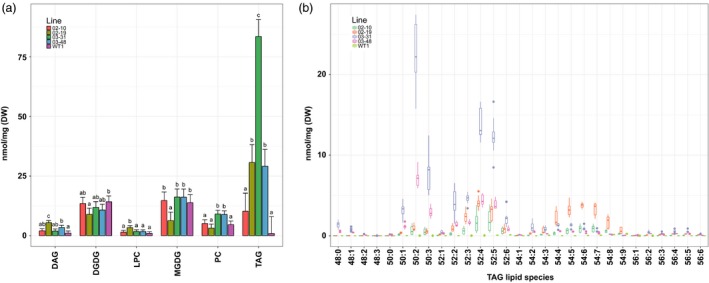
Lipidome analysis of wild‐type and transgenic *Sorghum bicolor* leaves at boot leaf stage. (a) Quantification of major neutral, phospholipid and galactolipid classes. Error bars indicate 95% confidence intervals of the LS means. Means sharing a letter are not significantly different (*P* < 0.01, Tukey‐adjusted) within the lipid class based on one‐way ANOVA. (b) Triacylglycerol (TAG) molecular species (number of carbon atoms: number of double bonds). Lines ‘02’ and ‘03’ were transformed with pOIL102+pOIL197 or pOIL103+pOIL197, respectively. DAG, diacylglycerol; DGDG, digalactosyldiacylglycerol; LPC, lysophosphatidylcholine; MGDG, monogalactosyldiacylglycerol; PC, phosphatidylcholine; TAG, triacylglycerol. Data are based on triplicate leaves from triplicate propagated tillers for each line.

Total lipid extracts from wild‐type and transgenic leaves sampled at boot leaf stage were subjected to LC‐MS to analyse different neutral and polar lipid classes in more detail. All four transgenic lines exhibited elevated TAG, amounting to a 100‐fold increase in line 03–31 compared to the wild‐type controls (Figure 4a). Small increases in PC were detected in both 03 transgenic lines, while levels of the plastidial galactolipids MGDG and DGDG were variable. Both LPC and DAG constituted minor lipid classes. TAG molecular species in lines 03–31 and 03–48 were enriched in palmitic acid and linoleic acid (Figure 4b). Major TAG species included TAG (50:2) and TAG (50:3) containing two palmitoyl chains and TAG (52:4) and TAG (52:5) consisting of palmitoyl and linoleoyl chains (Figure S5). Lines 02–10 and 02–19 exhibited distinctly different TAG profiles. Leaf tissues of both lines preferentially accumulated TAG consisting of one or more linoleoyl chains such as TAG (52:3‐5) and TAG (54:4‐8). The distinct differences in TAG profiles between the two transgenic populations were in line with earlier GC‐FID results (Figure S3).

Changes in TAG compositions were also reflected in the precursor DAG (Figure S6). Dominant DAG (34:2) and DAG (34:3) molecular species in lines 03–31 and 03–48 were enriched in palmitoyl chains while both 02 lines contained DAG molecules containing two C18 acyl chains (DAG 36:2‐6). Abundant eukaryotic galactolipid species such as MGDG (36:6) and DGDG (36:6) were either reduced or not significantly affected (Figure S6). Two prokaryotic galactolipid species, MGDG (34:3) and DGDG (34:2) were increased slightly in lines 03–31 and 03–48. The dominant prokaryotic DGDG species (34:3) was either unchanged or reduced in transgenic leaves. PC molecular species containing palmitoyl or linoleoyl chains including PC (34:1‐2) and PC (36:4) were elevated, particularly in lines 03–31 and 03–48 (Figure S6). Di‐palmitoyl PC (32:0) was increased in line 03–31, reflecting the higher levels of palmitoyl chains as detected by GC‐FID.

### TAG accumulation affects starch and amino acid content

Synthesis and accumulation of TAG in transgenic leaves of lines 03–31 and 03–48 had a negative impact on transitory starch levels which were reduced 7.4‐ and 15.3‐fold on average, respectively (Figure S7). Starch levels in leaves of both 02 lines were not significantly affected. Sucrose constituted the dominant leaf soluble sugar in all plants (Figure S8). Sucrose levels were twofold lower in line 03–48, while line 02–19 Line was comparable to the wild‐type control. Raffinose was reduced by 19.6‐fold in line 03–48, while monosaccharides such as glucose, fructose and galactose displayed smaller reductions.

A metabolite quantification by GC‐MS identified 36 compounds that were significantly different in leaves of wild‐type and transgenic lines (Table 2). Twenty metabolites were detected at higher levels in TAG‐accumulating leaves, including multiple amino acids, urea and the citric acid cycle (TCA) intermediate, α‐ketoglutarate. Several dicarboxylic acids, sugar alcohols, fructose, xylose and shikimate were amongst the metabolites that were less abundant in transgenic leaves. Principal component analysis revealed clear separations of both transgenic events and the wild‐type control (Figure S9).

**Table 2:** Metabolites displaying significant^a^ differences in accumulation in wild‐type vs. transgenic leaves (lines 02‐19 and 03‐48) at boot leaf stage

Metabolite	Trend	WT1/03‐48	*P*‐value	WT1/02‐19	*P*‐value	02‐19/03‐48	*P*‐value
D‐glucose	03‐48‐1 = 02‐19‐1> WT	0.621	0.004	0.599	0.028	1.037	0.844
Erythronic acid‐1,4‐lactone	03‐48‐1 = 02‐19‐1> WT	0.508	0.000	0.607	0.039	0.836	0.308
2‐oxo‐glutaric acid	03‐48‐1 = 02‐19‐1> WT	0.381	0.004	0.294	0.008	1.298	0.353
O‐acetylserine	03‐48‐1 = 02‐19‐1> WT	0.277	0.025	0.185	0.002	1.503	0.220
L‐glutamine	03‐48‐1 = 02‐19‐1> WT	0.112	0.002	0.144	0.033	0.782	0.536
Glyceric acid	03‐48‐1 > 02‐19‐1 > WT	0.365	0.000	0.599	0.011	0.609	0.001
Pyroglutamic acid	03‐48‐1 > 02‐19‐1 > WT	0.205	0.000	0.353	0.006	0.581	0.003
L‐threonine	03‐48‐1 > 02‐19‐1 > WT	0.150	0.002	0.346	0.001	0.435	0.022
L‐Serine	03‐48‐1 > 02‐19‐1 > WT	0.071	0.000	0.384	0.016	0.185	0.001
L‐Tyrosine	03‐48‐1 > 02‐19‐1 > WT	0.029	0.000	0.077	0.000	0.373	0.002
L‐cysteine	03‐48‐1 > 02‐19‐1 > WT	0.021	0.001	0.038	0.000	0.558	0.088
1,6‐anhydroglucose	03‐48‐1 > 02‐19‐1 > WT	0.021	0.003	0.122	0.037	0.169	0.009
L‐valine	03‐48‐1 > 02‐19‐1 > WT	0.012	0.004	0.031	0.003	0.396	0.055
L‐phenylalanine	03‐48‐1 > 02‐19‐1 > WT	0.009	0.001	0.041	0.000	0.209	0.006
Urea	03‐48‐1 > 02‐19‐1 > WT	0.007	0.003	0.015	0.005	0.499	0.111
L‐aspartic acid	03‐48‐1 > 02‐19‐1 > WT	0.007	0.002	0.024	0.000	0.293	0.015
Beta‐alanine	03‐48‐1 > 02‐19‐1 > WT	0.007	0.023	0.026	0.050	0.269	0.085
L‐methionine	03‐48‐1 > 02‐19‐1 > WT	0.004	0.005	0.021	0.001	0.216	0.019
L‐isoleucine	03‐48‐1 > 02‐19‐1 > WT	0.004	0.008	0.028	0.005	0.143	0.018
L‐Glutamic acid	03‐48‐1 > 02‐19‐1 > WT	0.001	0.000	0.001	0.002	0.525	0.015
Sorbose	WT > 02‐19‐1 > 03‐48‐1	4.232	0.000	1.898	0.000	2.230	0.000
Ribitol	WT > 02‐19‐1 > 03‐48‐1	3.275	0.000	1.499	0.000	2.185	0.000
2‐ketoglyconic acid	WT > 02‐19‐1 > 03‐48‐1	3.049	0.000	1.987	0.000	1.534	0.010
Shikimic acid	WT > 02‐19‐1 > 03‐48‐1	2.799	0.000	1.724	0.003	1.623	0.053
Fructose	WT > 02‐19‐1 > 03‐48‐1	1.990	0.000	1.456	0.013	1.366	0.079
Maleic acid	WT > 02‐19‐1 > 03‐48‐1	1.980	0.001	1.677	0.005	1.180	0.084
Xylitol	WT > 02‐19‐1 > 03‐48‐1	1.704	0.000	1.684	0.000	1.012	0.893
Maltose	WT > 02‐19‐1 > 03‐48‐1	1.439	0.000	1.371	0.001	1.050	0.578
Arabitol	WT > 02‐19‐1 > 03‐48‐1	1.402	0.001	1.409	0.001	0.995	0.957
Fumaric acid	WT > 02‐19‐1 > 03‐48‐1	1.322	0.028	1.293	0.037	1.022	0.633
Malic acid	WT > 02‐19‐1 > 03‐48‐1	1.319	0.001	1.719	0.000	0.767	0.016
Xylose	WT > 02‐19‐1 > 03‐48‐1	1.191	0.035	1.273	0.011	0.936	0.316
Malonic acid	WT > 02‐19‐1 > 03‐48‐1	0.790	0.005	1.980	0.000	0.399	0.000
Isoascorbic acid	WT > 02‐19‐1 > 03‐48‐1	0.694	0.001	1.499	0.004	0.463	0.000
Ribonic acid	WT > 02‐19‐1 > 03‐48‐1	0.597	0.000	1.425	0.004	0.419	0.000
Gulonic acid	WT > 02‐19‐1 > 03‐48‐1	0.485	0.000	1.473	0.001	0.330	0.000

a *P*‐values are based on a Welch *T*‐test and triplicate leaves from 2 to 3 propagated tillers for each line.

### Sorghum leaves accumulate TAG as cytosolic lipid droplets

**Figure 5:**
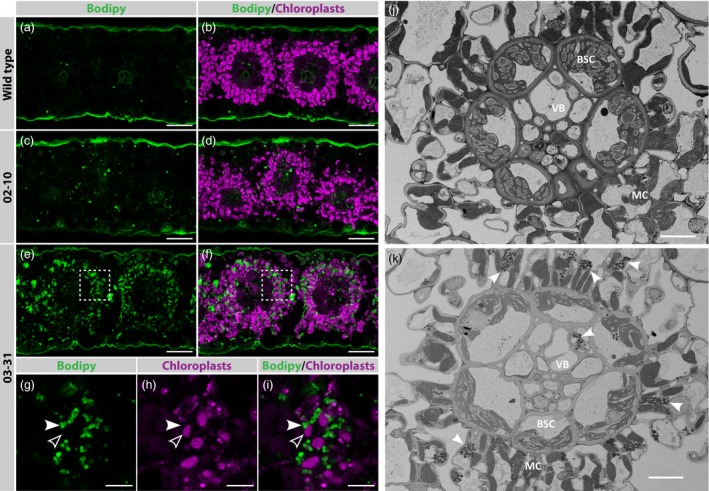
Accumulation of lipid droplets in *Sorghum bicolor* leaf mesophyll cells. (a–i) Confocal images showing oil content in fresh leaf cross sections of wild‐type (a,b) or transgenic tillers transformed with either pOIL102+pOIL197 (line 02–10; c,d) or pOIL103+pOIL197 (line 03–31; e–i). Lipid droplets were stained with Bodipy (green), while chloroplast autofluorescence was used to highlight the mesophyll chloroplasts (magenta). Panels g–i show a single plane taken in the region highlighted with a dashed box in e–f. (j–k) Images of wild‐type (j) and transgenic (line 03–31; h) leaf cross sections, collected by scanning electron microscopy. Cytosolic oil bodies and chloroplasts are marked by white and empty arrowheads, respectively. MC, mesophyll cell; BSC, bundle sheath cell; VB, vascular bundle. Scale bars correspond to 40 μm (a–f) and 10 μm (g–k).

To investigate in which cell types TAG accumulated in the 02–10 and 03–31 transgenic lines, thin fresh sections of leaves were harvested at boot leaf stage, stained with a dye marking neutral lipids, and imaged using confocal microscopy. In sorghum leaves, chloroplasts of bundle sheath and mesophyll cells have different auto‐fluorescing properties, allowing for easy distinction between the two cell types. In wild‐type sections, very few droplets were detected which localized in either cell type (Figure 5a,b; see Figure S10 for unstained sections). On the other hand, leaf sections of line 03–31 revealed an abundance of small lipid droplets that primarily accumulated in the cytosol of mesophyll cells (Figure 5e–i). This result was confirmed by scanning electron microscopy, which showed that small, densely packed lipid droplets accumulated preferentially in the cytosol of mesophyll cells in line 03–31 (Figure 5j and k). This distribution pattern likely reflects the tissue specificity of the PEPC promoter used to generate this particular transgenic line. Starch granules in bundle sheath cells of line 03–31 were smaller in size compared to the wild‐type control (Figure 5j and k). Finally, fresh leaf sections of line 02–10 revealed an intermediate number of lipid droplets in both mesophyll and bundle sheath cells (Figure 5c and d).

## Discussion

In this study, we relied on co‐transformation and lipid analysis of primary sorghum transformants as a means to rapidly screen different gene and promoter combinations. Co‐expression of *WRI1* and *DGAT2a* yielded up to 8.4% TAG and 9.9% TFA in flag leaf tissue during seed setting. Accumulation of TAG was visible as oil droplets throughout the leaf mesophyll cells. The levels achieved in this study are the highest reported in vegetative tissues of a C_4_ crop to date. Although absolute TAG levels in sugarcane were much lower compared to our results in sorghum, relative increases in both species were remarkably similar (95‐ and 100‐fold). As suggested by Zale *et al*. (2016), basal TAG metabolic rates might be different between crop species and therefore affect absolute increases achieved via combinatorial metabolic engineering. Transformation of separate *WRI1* and *DGAT2a* expression cassettes revealed a synergistic effect on TAG content when both transgenes were co‐expressed. Similar observations have been reported in *A. thaliana*, tobacco and sugarcane, suggesting both genes can act as key drivers for TAG biosynthesis in both dicot and monocot species (Kelly *et al*., 2013; Vanhercke *et al*., 2013; Zale *et al*. 2016).

We compared two different monocot promoters as previous studies in tobacco and *A. thaliana* had suggested promoter strength to be critical when co‐expressing *WRI1* and *DGAT2a* transgenes (Vanhercke *et al*., 2014; Zhai *et al*., 2017). Unlike tobacco and *A. thaliana*, it appears that at least sugarcane and sorghum can tolerate strong constitutive promoters to drive combined *WRI1* and *DGAT* expression. Although the use of the two different promoters resulted in comparable TAG levels, we did observe differences in the fatty acid profiles and lipidomes of the two transgenic sorghum populations. All transgenic plants exhibited a shift in fatty acid profile towards a lower degree of desaturation. This was mainly due to a reduction in α‐linoleic acid, similar to previous results obtained in tobacco (Vanhercke *et al*., 2014; Vanhercke *et al*., 2017). However, plants transformed with either the *Z. mays* Ubiquitin‐1 (pOIL102+pOIL197) or PEPC promoter driving *WRI1* expression (pOIL103+pOIL197) displayed distinct changes in the proportions of palmitic acid and linoleic acid in the TAG and TFA fractions. Linoleic acid was increased in transgenic 02 lines, whereas 03 plants typically contained higher palmitic acid levels. Consequently, both transgenic populations exhibited different ratios of C16 vs C18 fatty acids which were also clearly visible in the lipidome fingerprints as discussed below.

LC‐MS analysis of four selected transgenic lines containing two different genetic constructs revealed three major TAG groups. TAG molecular species composed of either one (52:3‐5) or two (50:2‐3) palmitoyl chains were enriched in 03 lines. On the other hand, TAG species in 02 transgenic plants typically contained either one palmitoyl group (52:3‐5) or three C18 acyl chains (54:4‐8). One possible explanation for the elevated levels of palmitic acid could be due to FatA up‐regulation as a result of the *WRI1* transgene overexpression (Maeo *et al*., 2009). The FatA thioesterase hydrolyses palmitoyl‐ACP, thereby releasing palmitic acid for subsequent incorporation into extraplastidial lipids. Increased DAG molecular species containing a palmitoyl chain (34:2‐3) likely serve as a substrate for the synthesis of TAG (50:2‐3). This would imply acylation of a second palmitoyl group at the *sn*‐3 position of the DAG backbone. Previous *in vitro* activity assays with *U. ramanniana* DGAT2a have shown considerable activity on DAG molecules containing shorter chain fatty acids as well as a lack of discrimination between DAG substrates containing C18 fatty acids (Lardizabal *et al*., 2001, 2008). Contrary to our observation, however, Oakes *et al*. (2011) found lower levels of palmitic acid in embryo oil following *U. ramanniana DGAT2a* overexpression in maize kernels. The different fatty acid profiles could be attributed to the nature of the tissue (source vs sink) and crop species. Whether or not *U. ramanniana* DGAT2a activity is responsible for the acylation of palmitic acid at the *sn*‐3 position of DAG resulting in di‐palmitoyl TAG requires further in‐depth biochemical studies using a variety of radiolabelled DAG and acyl‐CoA substrates. A similar reaction at the *sn*‐3 position of DAG containing two C18 groups (DAG 36:2‐5) would yield TAG (52:2‐5) in the 03 lines. Alternatively, acylation of DAG (34:2‐3) with a C18 group would lead to a similar outcome. The latter reaction might reflect endogenous phosphatidylcholine:diacylglycerol acyltransferase (PDAT) activity relying on increased levels of linoleic acid at the *sn*‐2 position of PC. In the 02 lines, elevated DAG (36:2‐6) species are likely the precursor for TAG consisting of three C18 acyl chains. Changes in PC followed the trend observed in DAG and TAG to some extent. Both DAG (32:2) and PC (32:2) species were highly increased in the 03 lines, hinting at a product–precursor relationship. Several enzymes are known to interconvert PC and DAG including phosphatidylcholine:diacylglycerol cholinephosphotransferase, cholinephosphotransferase and phospholipase C (Bates and Browse, 2012). Interestingly, in both 02 lines, DAG (36:6) was enriched, whereas PC (36:6) was depleted. This might reflect efficient flux of α‐linolenic acid from PC to DAG, followed by a bottleneck in the subsequent conversion to TAG. In the 03 lines, on the other hand, increased levels of palmitic acid might successfully compete for incorporation into PC and DAG, leading to lower levels of the 36:6 molecular species in both lipid classes.

Starch is a major carbon and energy storage form in leaves and some source tissues such as tubers and therefore expected to compete with lipids for photosynthetic carbon. Previous attempts at engineering elevated levels of storage lipids in a variety of species and tissues did negatively impact transitory or storage starch metabolism (Zale *et al*., 2016; Vanhercke *et al*., 2014; Hofvander *et al*., 2016; Liu *et al*., 2017; Mitchell *et al*., 2017; Vanhercke *et al*., 2017). Quantification of starch levels in transgenic sorghum leaves showed a distinct difference between the two transgenic populations. Transgenic 02 lines did not differ from wild‐type plants in their starch content. On the other hand, 03 lines were characterized by a reduction in transitory starch levels. GC‐MS analysis of soluble sugars revealed a similar trend with major mono‐, di‐ and trisaccharides reduced in leaves of 03 plants. Previous work in C_4_ plants has highlighted bundle sheath cells as the exclusive site of starch accumulation, whereas sucrose synthase activity in sorghum leaves was more pronounced in mesophyll cells (Lunn and Furbank, 1997). The inverse relationship between carbohydrate and storage lipid metabolism in the 03 lines and the occurrence of lipid droplets preferentially accumulating in the mesophyll cells suggests a reallocation of carbon into storage lipids as a result of mesophyll‐specific *WRI1* expression.

A wider metabolite comparison between wild‐type and transgenic leaves showed increased abundance of 11 amino acids. Urea, an intermediate of arginine catabolism (Witte, 2011), was also detected at elevated levels in transgenic sorghum leaves. Increased levels of amino acids have also been observed in transgenic potato tubers and maize kernels, overexpressing one or more genes involved in TAG biosynthesis (Mitchell *et al*., 2017; Pouvreau *et al*., 2011). In addition, heterologous *WRI1* expression in potato tubers has been reported to affect expression of genes involved in amino acid metabolism (Grimberg *et al*., 2015). Overall, the changes in amino acid content could be a direct result of *WRI1* overexpression, caused by changes in glycolytic and citric acid cycle (TCA) metabolic fluxes or due to indirect adjustments to the carbon and nitrogen metabolism as suggested by Pouvreau *et al*. (2011). Phenylalanine, valine, aspartate, threonine, isoleucine, methionine and tyrosine were amongst the amino acids that were increased in transgenic sorghum leaves. These amino acids originate from either pyruvate or phosphoenolpyruvate which also serve as precursors for lipid biosynthesis. Their elevated contents could reflect increased flux through the glycolytic pathway for subsequent *de novo* fatty acid synthesis. Moreover, phenylalanine and tyrosine are formed via the shikimate pathway which also provides precursors of other compounds including phenylpropanoids such as lignin. Hemicellulose, another cell wall component, consists of a variety of polysaccharides including xylose (Kumar *et al*., 2016). The effect of elevated levels of both amino acids in combination with reduced contents of shikimate and xylose on secondary cell wall composition in oil‐accumulating transgenic sorghum might have important implications for bioethanol production from left‐over biomass after TAG extraction. Amongst the TCA dicarboxylic acids, α‐ketoglutarate levels were increased in transgenic leaves while both malate and fumarate were reduced. This could reflect changes to TCA flux as a consequence of altered N‐metabolism since α‐ketoglutarate is involved in the synthesis of glutamate (Sweetlove *et al*., 2010). Sorghum and other plants belonging to the NADP‐malic C_4_ group rely on malate for the transport of photosynthetic carbon between leaf mesophyll and bundle sheath cells. Lower malate levels in mesophyll cells as a result of increased demand for lipid and possibly amino acid synthesis are expected to directly impact carbon fixation in the bundle sheath cells, explaining the reduction in overall starch levels.

Low *Oleosin‐L* transcript levels were detected in the majority of the transgenic lines. In addition, lipid accumulation was not correlated with oleosin expression and therefore most likely attributable to *WRI1* and *DGAT2a* expression only. Likely oil body coating is suboptimal within leaf cells of the transgenic sorghum lines in this study due to low *Oleosin‐L* expression. Oil body proteins such as oleosin are known to stabilize oil droplet integrity and reduce TAG turnover (Fan *et al*., 2013b; Winichayakul *et al*., 2013). A futile TAG cycle occurring in the current transgenic lines would limit the accumulation of oil, particularly in senescing leaves during later stages of plant development. Overexpression of alternative functional oil body proteins such as caleosin or lipid droplet‐associated proteins are possible ways to improve lipid droplet stability and further elevate TAG levels in sorghum (Gidda *et al*., 2016; Shimada *et al*., 2014). Alternatively, silencing of the *SDP1* lipase or overexpression of the *LEC2* master regulator of embryogenesis has also been shown to partly overcome TAG turnover in nonseed tissues (Kelly *et al*., 2013; Vanhercke *et al*., 2017). Finally, overall lipid content in transgenic sorghum stems was much lower compared to leaves. Unlike leaves, stems are major sink tissues in sweet sorghum as they accumulate high levels of fermentable sugars. Improving the energy density in stem tissues of C_4_ crops such as sweet sorghum will likely require genetic manipulation of additional genes such as those implicated in sugar transport and storage in stem parenchyma cells in combination with carefully selected stem‐specific promoters (Bihmidine *et al*., 2016; Qazi *et al*., 2012).

The high biomass yields of some cultivars and high leaf‐to‐stem ratio of subspecies such as *S. bicolor drumondii* (sudangrass) make sorghum an ideal and versatile target crop for the engineering of extractable levels of plant oils throughout the entire vegetative biomass. The work presented here provides a first step towards the development of sorghum as a dedicated oil or dual‐purpose crop that has the potential to outcompete current oilcrops for future food, feed, oleochemical and biofuel needs.

## Material and methods

### Plant expression vectors

We designed three expression constructs for biolistic co‐transformation of *Sorghum Bicolor* Tx430. A synthetic fragment containing the *Z. mays WRI1* gene (Shen *et al*., 2010a), codon optimized for expression in *Triticum aestivum*, followed by the *Glycine max* Lectin terminator was subcloned into the *Asc*I and *Not*I sites of pJP3343, a pORE04‐based binary expression vector (Coutu *et al*., 2007). The resulting vector was designated pOIL100. The *Z. mays* Ubiquitin‐1 promoter (Christensen *et al*., 1992) was amplified from pUKN (Liu and Godwin, 2012) and subcloned as a *Hin*dIII‐*Avr*II fragment into the *Hin*dIII‐*Xba*I sites of pOIL100, resulting in pOIL102. The *Z. mays* PEPC promoter (Matsuoka and Minami, 1989) was synthesized and subcloned into pOIL100 using the *Hin*dIII and *Nco*I restriction sites, resulting in pOIL103.

The *Oryza sativa* Actin‐1 promoter (McElroy *et al*., 1990) was amplified from pZLAct1cas (Dr. Zhongyi Li, CSIRO) and cloned as an *Xho*I‐*Cla*I fragment into the *Spe*I‐*Cla*I sites of pORE04, resulting in pOIL094. To this end, *Xho*I and *Spe*I restriction sites were treated with T4 DNA polymerase to generate blunt ends prior to digestion with *Cla*I. Plasmid pOIL095 was subsequently generated by inserting the gene coding for the *Sesamum indicum* Oleosin L‐isoform (Tai *et al*., 2002) into the *Kpn*I site of pOIL094. The *Umbelopsis ramanniana DGAT2a* gene (Lardizabal *et al*., 2008) was codon optimized for expression in *Triticum aestivum*, synthesized as a *Sma*I‐*Kpn*I fragment and subcloned downstream of the *Z. mays* Ubiquitin‐1 promoter in pZLUbi1casNK (Dr. Zhongyi Li, CSIRO), yielding pOIL093. Next, pOIL134 was obtained by subcloning the entire DGAT2a expression cassette from pOIL093 into the *Not*I site of pOIL095. The *Z. mays* Ubiquitin‐1 promoter sequence followed by the *NPTII* gene was removed from pUKN as a *Hin*dIII‐*Sma*I fragment and cloned into the *Asc*I‐*Hin*dIII sites of pJP3343. The *Asc*I in pJP3343 was first treated with T4 DNA polymerase prior to *Hin*dIII digestion and subsequent ligation. The resulting vector pOIL196 was digested with *Hin*dIII, followed by treatment with T4 DNA polymerase and digestion with *Age*I. The resulting 3358 bp fragment was subcloned into pOIL134 at the *Zra*I‐*Age*I sites. The resulting vector was designated pOIL197. The pOIL197 subcloning strategy resulted in an additional Nopaline synthase polyadenylation signal in‐between the *Z. mays* Ubiquitin‐1 and *O. sativa* Actin‐1 promoter sequences. Gene syntheses were done by GeneArt (Thermo Fisher Scientific).

### *S. bicolor* transformation

Differentiating embryogenic callus was generated from grain sorghum inbred line Tx430 and used as explant for genetic transformation of sorghum and putative transgenic plants were regenerated as described by Belide *et al*. (2017). Negative wild‐type controls were taken through tissue culture without antibiotic selection. Transgenic plants were grown in the glasshouse under 28 ± 1 °C/20 ± 1 °C and natural light. Additional transformations of pOIL102 or pOIL197 were undertaken at the University of Queensland essentially as described (Liu and Godwin, 2012) and subsequently grown alongside the other transgenic lines in the glasshouse. Care was taken to cut back emerging tillers during the sampling period to maximize carbon availability in leaves on the main culm. Following final sampling, tillers were allowed to develop for subsequent propagation.

### Copy number and gene expression analyses

Transgene copy numbers were determined by digital droplet PCR (ddPCR) essentially as described in Belide *et al*. (2017) using *S. bicolor enol2* reference gene (Casu *et al*., 2012). For gene expression analysis by ddPCR, total RNA from leaf tissue was extracted using RNA Easy plant kit (Qiagen). Total RNA (2 μg) was treated with RQ1 RNase‐Free DNase (Promega), followed by reverse transcription using SuperScript III VILO Master Mix (Invitrogen). Each 25 μL reaction volume contained 12.5 μL of ddPCR Supermix master mix, 400 nm of each primer, 200 nm of the probe and 1 μL cDNA. Oil droplet generation, PCR amplification and data analysis were done as described (Vanhercke *et al*., 2017). The *S. bicolor* actin gene served as reference for gene expression analysis (Shen *et al*., 2010a, b). Primer and probe sequences used in ddPCR reactions are detailed in Table S1.

### Total lipid and TAG quantification

Total fatty acids (TFA) were extracted from 100 mg freeze‐dried tissue as previously described (Vanhercke *et al*., 2013). Solvent of the lipid extract was evaporated under a N_2_ flow and total lipids were resuspended in 2 μL chloroform per mg leaf DW. Total lipids were separated by thin layer chromatography in hexane:diethylether:acetic acid (70:30:1, v:v:v) as previously described (Vanhercke *et al*., 2013). Fatty acid methyl esters (FAME) of total lipids (15 mg leaf DW) or TAG (30 mg leaf DW) were prepared in 750 μL 1N methanolic‐HCl in the presence of triheptadecanoin (Nuchek Prep, Inc.) as an internal standard (Vanhercke *et al*., 2013). FAME analysis was performed on an Agilent 7890A gas chromatography system with flame ionization detection (GC‐FID). Chromatograms were recorded and peaks were identified against retention times of authentic FAME standards (NuChek Prep, Inc).

### LC‐MS analysis

Sorghum leaf samples were first analysed qualitatively on an Agilent 6550 Q‐TOF to identify lipid species, and quantitative multiple reaction monitoring (MRM) analysis was conducted on an Agilent 6490 triple quadrupole mass spectrometer. Chloroform extracts containing lipids were dried under nitrogen and resuspended in chloroform to 100 mg/mL, then diluted to 1 mg/mL in butanol:methanol (1:1, v:v) prior to analysis by liquid chromatography–mass spectrometry (LC‐MS). Lipids were separated on a Waters BEH C8 (100 mm × 2.1 mm, 2.7 μm) with an Agilent 1290 series operating a binary gradient flow rate of 0.2 mL/min. The mobile phases were (A) H_2_O:acetonitrile (10:90, v:v) with 10 mm ammonium formate and 0.2% formic acid and (B) H_2_O:acetonitrile:isopropanol (5:15:80, v:v:v) containing 10 mm ammonium formate and 0.2% formic acid. The gradient was held at 1% mobile phase (B) for 2 min before being raised to 20% (B) at 5 min, 60% (B) at 8 min, 70% (B) at 12 min and final increase to 90% (B) at 14 min before re‐equilibration to 1% (B). The eluent was introduced into an Agilent Jet Stream source with ionization conditions as follows: nebulizer at 45 psi, gas temperature and flow rates set at 250 °C and 14 L/min, sheath gas and flow at 250 °C and 11 L/min, capillary and nozzle voltages set at 3000 V and 100 V, respectively. The positive ion ammonium adducts of monogalactosyldiacylglycerol (MGDG), digalactosyldiacylglycerol (DGDG), diacylglycerol (DAG) and TAG lipid species were fragmented using a collision energy of 28 V for all lipid classes except for DAG where a collision energy of 14 V was used. Multiple reaction monitoring (MRM) lists were based on lipid species detected by Q‐TOF and the neutral loss of the following major fatty acids: C16:0, C16:3, C18:0, C18:1, C18:2 and C18:3. The positive ion phosphatidylcholine (PC) and lysophosphatidylcholine (LPC) species hydrogen adducts were quantified by the characteristic 184 *m/z* phosphatidyl head group.

External standards were purchased from Avanti Polar Lipids (PC 36:2 18:1/18:1, MGDG 34:6 16:3/18:3, DGDG 34:3 16:0/18:3, DAG 36:2 18:1/18:1, TAG 54:3 18:1/18:1/18:1) and standard curves ranging from 0.001 μg/mL to 100 μg/mL were used to quantify all lipid species within each class. Peak integration and initial analysis were conducted using MassHunter Quantitative software B.07.00 (Agilent Technologies) and exported for further data analysis using the dplyr and ggplot1 packages from R v3.4.0 using RStudio v1.0.143 (Horton and Kleinman, 2015).

### Metabolite analyses

Polar secondary metabolites were first extracted by homogenizing frozen leaf tissue in 5× volume per tissue weight of methanol:H_2_O (20:3, v:v) containing 8.7 μg/mL sorbitol. The homogenate was centrifuged at 10 000 ***g*** for 5 min to remove solid matter and the supernatant was stored at −80 °C until further analysis. Next, 30 μL of the supernatant was dried down in a vacuum concentrator and methoxylated with 10 μL of 20 mg/mL methoxyamine hydrochloride in pyridine at 37 °C for 90 min, followed by silylation with 15 μL of N‐methyl‐N‐(trimethylsilyl)‐trifluoroacetamide (Sigma‐Aldrich) at 37 °C for 30 min. Prior to GC‐MS analysis, 5 μL of an n‐alkane mix containing n‐dodecane, n‐hexadecane, n‐eicosane, n‐heneicosane, n‐docosane and n‐pentacosane was added to each derivatized sample at a final concentration of 0.029% each (v:v or w:v for n‐alkanes that were liquid or solid at ambient temperature, respectively). One μL of the derivatized sample was analysed by GC‐MS according to Mitchell *et al*. (2017). MetabolomeExpress was used to process raw GC‐MS data and conduct statistical analyses (Carroll *et al*., 2010). Metabolites were identified by mass spectral data as well as retention index information using the public metabolome library in MetabolomeExpress, the Golm metabolome database (Hummel *et al*., 2007) and NIST 14 mass spectral library (http://chemdata.nist.gov/dokuwiki/doku.php?id=chemdata:start). D‐(‐)‐fructose, D‐(+)‐glucose, D‐(+)‐galactose, sucrose and D‐(+)‐raffinose (Sigma‐Aldrich) were derivatized as per above and subjected to GC‐MS to determine retention times. Peak areas of the sugars in the sorghum samples with the corresponding retentions times were extracted from the total ion chromatograms using MassHunter Qualitative B.07.00 (Agilent Technologies) and normalized against the extracted peak area of sorbitol. Starch content in leaf tissue at the end of the day was quantified essentially as previously described (Vanhercke *et al*., 2017).

### Confocal laser‐scanning microscopy

Flag leaves of re‐established side tillers from transgenic *S. bicolor* plants were harvested at the beginning of flowering and kept on ice until sections were prepared for imaging. Fresh, thin hand sections were stained for 10 min with a solution of 50 mm PIPES pH7 supplemented with 2 μg/mL of BODIPY 505–515 (4,4‐Difluoro‐1,3,5,7‐Tetramethyl‐4‐Bora‐3a,4a‐Diaza‐s‐Indacene, Thermo Fisher Scientific). They were then rinsed in a solution of PIPES pH7 and imaged right away. Control sections were placed directly in 50 mm PIPES pH 7 for 10 min before being mounted on slides and imaged to establish background fluorescence of the tissue.

All samples were imaged with a confocal laser‐scanning microscope (Leica TCS SP8) equipped with a white light laser and a 40× water immersion objective ([NA] = 1.1), and controlled by the LAS X software (Leica Microsystems). Imaging was done in a sequential manner: BODIPY was excited at 505 nm and its emission was collected at 520‐540 nm, while in a separate track, chloroplasts were excited at 633 nm and their autofluorescence was collected at 650–690 nm. Maximum projections were generated with the LAS X software.

### Scanning electron microscopy

For ultrastructural analysis, harvested sorghum leaf tissues were cut into 1 mm × 2 mm size samples and fixed in 2.5% glutaraldehyde (SIP Supplies) and 4% paraformaldehyde (SPI Supplies) in 0.1 m phosphate buffer (pH 7.2) and subsequently post‐fixed in 1% osmium tetroxide (SPI Supplies) at room temperature for 4 h. The fixed samples were dehydrated in a graded ethanol series and infiltrated with LR white acrylic resin (medium grade, Sigma‐Aldrich). Subsequently, polymerization occurred at 60 °C in a vacuum oven under nitrogen. Following thin sectioning on a UC7 ultramicrotome (Leica) onto indium tin oxide, SPI Supplies) coverslips, the 300 nm thin sections were poststained with uranyl acetate (1% aqueous) and lead citrate (Reynolds). The samples were examined in a scanning electron microscope (ZEISS UltraPlus FESEM) at an accelerating voltage of 3 kV using the back‐scattered detector. The contrast was inversed for all captured images.

## Author contributions

T.V., S.P.S. and J.R.P. designed research. T.V., S.B., M.C.T., A.E.T., S.O., V.R., Q.L., M.M., P.S., I.V., L.M., C.B., A.M., L.Z., N.N., D.H., B.D., G.L., I.D.G., J.L. and M.R. performed research. T.V., S.B., M.C.T., A.E.T., S.O., V.R., Q.L., M.M., P.S., X.R.Z., S.P.S. and J.R.P. analysed data. T.V., S.B., M.C.T., S.O. and V.R. wrote the manuscript.

## Supporting information


**Figure S1** Total lipids (TFA) and triacylglycerol (TAG) contents on a dry weight basis (DW) in leaves of selected transgenic *Sorghum bicolor* at boot leaf stage. Sorghum was transformed with pOIL102, pOIL197, pOIL102+pOIL197 or pOIL103+pOIL197. Selected primary transformants were re‐established from side‐tillers obtained from selected independent primary transformants. Means and standards deviations are based on triplicate leaves for each transgenic line.
**Figure S2** Total lipid (TFA) composition and content on a dry weight basis (DW) in leaves of wild‐type and transgenic *Sorghum bicolor* at boot leaf stage. Only major fatty acids are shown. Triplicate tillers were propagated from four selected independent primary transgenic events, transformed either with pOIL102+pOIL197 (‘02’ events) or pOIL103+pOIL197 (‘03’ events). Means and standards deviations are based on triplicate leaves from each propagated tiller.
**Figure S3** Triacylglycerol (TAG) composition and content on a dry weight basis (DW) in leaves of wild‐type and transgenic *Sorghum bicolor* at boot leaf stage. Only major fatty acids are shown. Triplicate tillers were propagated from four selected independent primary transgenic events, transformed either with pOIL102+pOIL197 (‘02’ events) or pOIL103+pOIL197 (‘03’ events). Means and standards deviations are based on triplicate leaves from each propagated tiller.
**Figure S4** Total lipid (TFA) and triacylglycerol (TAG) contents (% dry weight) in leaf and stem tissues of wild‐type and transgenic *Sorghum bicolor* at seed setting stage. Transgenic ‘02’ and ‘03’ events were transformed with pOIL102+pOIL197 or pOIL103+pOIL197, respectively. Leaves from propagated tillers were numbered from top to bottom, starting from the flag leaf. Stem tissues were sampled from the bottom third part of the tiller. Values are based on single measurements.
**Figure S5** Fatty acid composition of selected TAG species in leaves of wild‐type and transgenic *Sorghum bicolor* at boot leaf stage. Triplicate tillers were propagated from four selected independent primary transgenic events, transformed either with pOIL102+pOIL197 (‘02’ events) or pOIL103+pOIL197 (‘03’ events). Individual TAG species are labelled based on the total number of carbon atoms and double bonds. Means and standards deviations are based on triplicate leaves from each propagated tiller.
**Figure S6** Diacylglycerol (DAG), digalactosyldiacylglycerol (DGDG), monogalacosyldiacylglycerol (MGDG) and phosphatidylcholine (PC) molecular species (number of carbon atoms: number of double bonds) in wild‐type and transgenic *Sorghum bicolor* leaves at boot leaf stage. Lines ‘02’ and ‘03’ were transformed with pOIL102+pOIL197 or pOIL103+pOIL197, respectively. Means and standards deviations are based on triplicate leaves from triplicate propagated tillers.
**Figure S7** Starch content (% dry weight) in leaves of wild‐type and transgenic *Sorghum bicolor* at boot leaf stage. Triplicate tillers were propagated from four selected independent primary transgenic events, transformed either with pOIL102+pOIL197 (‘02’ events) or pOIL103+pOIL197 (‘03’ events). Means and standards deviations are based on triplicate leaves from each propagated tiller.
**Figure S8** Relative contents of different soluble sugars in leaves of wild‐type and transgenic *Sorghum bicolor* at boot leaf stage. Transgenic lines 02–19 and 03–48 were transformed either with pOIL102+pOIL197 or pOIL103+pOIL197, respectively. Glucose and galactose were detected as two peaks (‘mp’, major product; ‘bp’, biproduct) with identical mass spectra. Means and standards deviations are based on triplicate leaves from 2 to 3 propagated tillers.
**Figure S9** Principle component analysis (PCA) of metabolites in wild‐type and transgenic *Sorghum bicolor* leaves at boot leaf stage. Wild‐type (open triangles), lines 02–19 (open circles) and 03–48 (closed circles) are clustered.
**Figure S10** Confocal images of unstained fresh *Sorghum bicolor* leaf cross‐sections of wild‐type (a,b) or propagated tillers transformed with either pOIL102+pOIL197 (c,d) or pOIL103+pOIL197 (e–f), showing that there was no autofluorescence in the Bodipy channel (green, a,c,e). Chloroplast autofluorescence was used to highlight the mesophyll chloroplasts (magenta, b,d,f,h,i). Scale bars: 40 μm.
**Table S1** Digital PCR primer and probe sequences.
**Table S2** Transgene copy numbers in *Sorghum bicolor* primary transformants.
**Table S3** Transgene expression levels in propagated tillers of selected lines normalized to *S. bicolor actin*.
