# Supplementary material for: Up‐regulation of lipid biosynthesis increases the oil content in leaves of Sorghum bicolor
Source: Plant Biotechnol J. 2018 Jul 13;17(1):220–32. doi: 10.1111/pbi.12959 (PMC6330533; doi:10.1111/pbi.12959)
Supplement: Supplementary file 1 — Figure S1 Total lipids (TFA) and triacylglycerol (TAG) contents on a dry weight basis (DW) in leaves of selected transgenic Sorghum bicolor at boot leaf stage. Sorghum was transformed with pOIL102, pOIL197, pOIL102+pOIL197 or pOIL103+pOIL197. Selected primary transformants were re‐established from side‐tillers obtained from selected independent primary transformants. Means and standards deviations are based on triplicate leaves for each transgenic line. Figure S2 Total lipid (TFA) composition and content on a dry weight basis (DW) in leaves of wild‐type and transgenic Sorghum bicolor at boot leaf stage. Only major fatty acids are shown. Triplicate tillers were propagated from four selected independent primary transgenic events, transformed either with pOIL102+pOIL197 (‘02’ events) or pOIL103+pOIL197 (‘03’ events). Means and standards deviations are based on triplicate leaves from each propagated tiller. Figure S3 Triacylglycerol (TAG) composition and content on a dry weight basis (DW) in leaves of wild‐type and transgenic Sorghum bicolor at boot leaf stage. Only major fatty acids are shown. Triplicate tillers were propagated from four selected independent primary transgenic events, transformed either with pOIL102+pOIL197 (‘02’ events) or pOIL103+pOIL197 (‘03’ events). Means and standards deviations are based on triplicate leaves from each propagated tiller. Figure S4 Total lipid (TFA) and triacylglycerol (TAG) contents (% dry weight) in leaf and stem tissues of wild‐type and transgenic Sorghum bicolor at seed setting stage. Transgenic ‘02’ and ‘03’ events were transformed with pOIL102+pOIL197 or pOIL103+pOIL197, respectively. Leaves from propagated tillers were numbered from top to bottom, starting from the flag leaf. Stem tissues were sampled from the bottom third part of the tiller. Values are based on single measurements. Figure S5 Fatty acid composition of selected TAG species in leaves of wild‐type and transgenic Sorghum bicolor at boot leaf stage. Triplicate t [file PBI-17-220-s001.docx]

**Supplementary Table 1.** Digital PCR primer and probe sequences.

| **Target gene** | **Primer/Probe** | **Name** | **Sequence (5’ - 3’)** |
| --- | --- | --- | --- |
| *S. bicolor* *Enolase2* | Sense primer | Can169 | tgaggacccttttgatcagg |
| *S. bicolor* *Enolase2* | Antisense primer | Can170 | caagccttcttgccaatagc |
| *S. bicolor* *Enolase2* | Probe | Sb-Enol-P2 | tggagttcatgggcatcattgca |
| *S. bicolor Actin* | Sense primer | Can247 | cgcggctactccttcactac |
| *S. bicolor Actin* | Antisense primer | Can248 | gcgtagtccagggcaatgt |
| *S. bicolor Actin* | Probe | Sb-Actin-P3 | gtcgtagtccagggcaatgt |
| *Z. mays WRI1* | Sense primer | Can251 | cgcagatcctctggattctc |
| *Z. mays WRI1* | Antisense primer | Can252 | gatcctagcctcccaccttc |
| *Z. mays WRI1* | Probe | Maize WRI-P1 | tggagttcatgggcatcattgca |
| *U. ramanniana DGAT2* | Sense primer | Can261 | aggtgcctctgagtccttga |
| *U. ramanniana DGAT2* | Antisense primer | Can262 | ctagcaccagtctgcacagc |
| *U. ramanniana DGAT2* | Probe | UrDGAT2a-P3 | acgccagaccaggagtgatgga |
| *S. indicum Oleosin-L* | Sense primer | Can265 | atcaacttgagtccgccaag |
| *S. indicum Oleosin-L* | Antisense primer | Can266 | ccactggttgttgggagaat |
| *S. indicum Oleosin-L* | Probe | SiOleosin-P2 | aaggcccgcgagatgaagga |

**Supplementary Table 2.** Transgene copy numbers in *Sorghum bicolor* primary transformants.

| **Line** | **Construct(s)** | **WRI1** | **DGAT2** | **Oleosin-L** |
| --- | --- | --- | --- | --- |
| **WT87** |  | 0.0 | 0.0 | 0.0 |
| **WT88** |  | 0.0 | 0.0 | 0.0 |
| **01-1** | pOIL102 | 0.0 | 0.0 | 0.0 |
| **01-2** | pOIL102 | 2.8 | 0.0 | 0.0 |
| **01-3** | pOIL102 | 2.1 | 0.0 | 0.0 |
| **01-4** | pOIL102 | 0.0 | 0.0 | 0.0 |
| **01-5** | pOIL102 | 0.0 | 0.0 | 0.0 |
| **01-6** | pOIL102 | 2.7 | 0.0 | 0.0 |
| **01-7** | pOIL102 | 2.2 | 0.0 | 0.0 |
| **01-8** | pOIL102 | 0.0 | 0.0 | 0.0 |
| **01-9** | pOIL102 | 0.0 | 0.0 | 0.0 |
| **197-8** | pOIL197 | 0.0 | 1.1 | 0.4 |
| **197-9** | pOIL197 | 0.0 | 1.9 | 1.5 |
| **197-10** | pOIL197 | 0.0 | 9.0 | 10.0 |
| **197-11** | pOIL197 | 0.0 | 6.0 | 8.3 |
| **197-13** | pOIL197 | 0.0 | 48.0 | 38.0 |
| **197-14** | pOIL197 | 0.0 | 7.1 | 5.7 |
| **197-15** | pOIL197 | 0.0 | 7.3 | 8.5 |
| **197-16** | pOIL197 | 0.0 | 7.3 | 3.1 |
| **197-17** | pOIL197 | 0.0 | 4.5 | 4.5 |
| **197-18** | pOIL197 | 0.0 | 21.3 | 1.8 |
| **197-19** | pOIL197 | 0.0 | 8.3 | 2.5 |
| **197-20** | pOIL197 | 0.0 | 2.4 | 1.0 |
| **197-21** | pOIL197 | 0.0 | 1.9 | 1.6 |
| **197-22** | pOIL197 | 0.0 | 2.3 | 2.4 |
| **197-23** | pOIL197 | 0.0 | 6.4 | 1.2 |
| **197-32** | pOIL197 | 0.0 | 4.3 | 1.0 |
| **197-33** | pOIL197 | 0.0 | 1.2 | 0.0 |
| **197-34** | pOIL197 | 0.0 | 1.1 | 4.0 |
| **197-35** | pOIL197 | 0.0 | 4.9 | 4.0 |
| **197-36** | pOIL197 | 0.0 | 1.3 | 0.0 |
| **197-38** | pOIL197 | 0.0 | 2.6 | 1.6 |
| **197-39** | pOIL197 | 0.0 | 1.4 | 0.0 |
| **197-40** | pOIL197 | 0.0 | 0.0 | 16.4 |
| **02-1** | pOIL102+pOIL197 | 2.8 | 1.5 | 3.3 |
| **02-2** | pOIL102+pOIL197 | 2.1 | 4.0 | 2.5 |
| **02-4** | pOIL102+pOIL197 | 7.0 | 8.2 | 1.9 |
| **02-5** | pOIL102+pOIL197 | 4.1 | 4.9 | 3.4 |
| **02-6** | pOIL102+pOIL197 | 8.9 | 6.0 | 4.7 |
| **02-7** | pOIL102+pOIL197 | 2.8 | 1.5 | 2.5 |
| **02-8** | pOIL102+pOIL197 | 2.1 | 3.4 | 2.4 |
| **02-9** | pOIL102+pOIL197 | 12.0 | 5.9 | 7.0 |
| **02-10** | pOIL102+pOIL197 | 4.3 | 10.7 | 13.9 |
| **02-11** | pOIL102+pOIL197 | 4.1 | 4.5 | 3.9 |
| **02-12** | pOIL102+pOIL197 | 21.3 | 13.3 | 12.9 |
| **02-13** | pOIL102+pOIL197 | 3.1 | 3.7 | 3.2 |
| **02-14** | pOIL102+pOIL197 | 2.4 | 1.7 | 2.4 |
| **02-16** | pOIL102+pOIL197 | 8.0 | 13.0 | 9.4 |
| **02-18** | pOIL102+pOIL197 | 1.4 | 1.4 | 2.5 |
| **02-19** | pOIL102+pOIL197 | 2.6 | 5.0 | 3.5 |
| **02-21** | pOIL102+pOIL197 | 2.0 | 6.6 | 6.0 |
| **02-23** | pOIL102+pOIL197 | 0.0 | 0.0 | 0.0 |
| **02-24** | pOIL102+pOIL197 | 2.0 | 4.8 | 1.4 |
| **02-25** | pOIL102+pOIL197 | 2.6 | 6.3 | 2.7 |
| **02-27** | pOIL102+pOIL197 | 0.0 | 0.0 | 0.0 |
| **02-28** | pOIL102+pOIL197 | 12.5 | 12.5 | 14.8 |
| **02-29** | pOIL102+pOIL197 | 0.0 | 0.0 | 0.0 |
| **02-30** | pOIL102+pOIL197 | 2.2 | 4.4 | 3.0 |
| **02-31** | pOIL102+pOIL197 | 3.8 | 1.1 | 3.0 |
| **02-33** | pOIL102+pOIL197 | 4.1 | 2.0 | 3.2 |
| **02-34** | pOIL102+pOIL197 | 1.5 | 2.2 | 3.1 |
| **02-35** | pOIL102+pOIL197 | 3.1 | 0.0 | 1.1 |
| **02-36** | pOIL102+pOIL197 | 0.5 | 3.0 | 1.7 |
| **02-37** | pOIL102+pOIL197 | 0.0 | 0.0 | 0.0 |
| **02-38** | pOIL102+pOIL197 | 5.6 | 11.3 | 11.3 |
| **02-39** | pOIL102+pOIL197 | 1.2 | 2.2 | 1.4 |
| **02-40** | pOIL102+pOIL197 | 1.2 | 0.0 | 6.7 |
| **02-41** | pOIL102+pOIL197 | 0.0 | 2.2 | 1.2 |
| **02-42** | pOIL102+pOIL197 | 1.8 | 3.5 | 8.1 |
| **02-43** | pOIL102+pOIL197 | 0.0 | 2.3 | 4.3 |
| **02-44** | pOIL102+pOIL197 | 1.3 | 2.1 | 1.1 |
| **03-1** | pOIL103+pOIL197 | 2.3 | 2.2 | 2.3 |
| **03-2** | pOIL103+pOIL197 | 2.4 | 2.7 | 2.5 |
| **03-4** | pOIL103+pOIL197 | 4.0 | 1.0 | 6.2 |
| **03-5** | pOIL103+pOIL197 | 1.0 | 2.9 | 4.0 |
| **03-7** | pOIL103+pOIL197 | 5.5 | 1.2 | 3.9 |
| **03-8** | pOIL103+pOIL197 | 2.5 | 2.5 | 2.2 |
| **03-10** | pOIL103+pOIL197 | 37.0 | 17.0 | 18.3 |
| **03-11** | pOIL103+pOIL197 | 60.0 | 19.0 | 21.0 |
| **03-16** | pOIL103+pOIL197 | 0.7 | 0.0 | 0.0 |
| **03-17** | pOIL103+pOIL197 | 5.8 | 1.7 | 1.6 |
| **03-18** | pOIL103+pOIL197 | 22.0 | 12.0 | 24.0 |
| **03-19** | pOIL103+pOIL197 | 9.7 | 3.2 | 9.0 |
| **03-20** | pOIL103+pOIL197 | 0.7 | 2.6 | 3.9 |
| **03-21** | pOIL103+pOIL197 | 14.8 | 8.1 | 15.8 |
| **03-22** | pOIL103+pOIL197 | 10.4 | 12.0 | 27.0 |
| **03-23** | pOIL103+pOIL197 | 4.4 | 2.4 | 1.6 |
| **03-25** | pOIL103+pOIL197 | 8.9 | 8.9 | 10.3 |
| **03-26** | pOIL103+pOIL197 | 8.6 | 2.3 | 6.9 |
| **03-27** | pOIL103+pOIL197 | 0.0 | 0.0 | 0.0 |
| **03-28** | pOIL103+pOIL197 | 15.0 | 9.6 | 8.6 |
| **03-29** | pOIL103+pOIL197 | 19.0 | 11.1 | 14.8 |
| **03-31** | pOIL103+pOIL197 | 31.0 | 8.6 | 6.1 |
| **03-32** | pOIL103+pOIL197 | 36.0 | 8.2 | 17.0 |
| **03-33** | pOIL103+pOIL197 | 4.2 | 16.0 | 15.0 |
| **03-34** | pOIL103+pOIL197 | 6.6 | 4.1 | 12.0 |
| **03-36** | pOIL103+pOIL197 | 2.4 | 2.6 | 4.5 |
| **03-40** | pOIL103+pOIL197 | 6.6 | 6.5 | 9.5 |
| **03-41** | pOIL103+pOIL197 | 7.9 | 6.7 | 6.6 |
| **03-45** | pOIL103+pOIL197 | 10.0 | 9.1 | 13.7 |
| **03-46** | pOIL103+pOIL197 | 6.6 | 4.9 | 7.7 |
| **03-47** | pOIL103+pOIL197 | 4.4 | 1.2 | 1.3 |
| **03-48** | pOIL103+pOIL197 | 26.0 | 7.2 | 4.1 |
| **03-49** | pOIL103+pOIL197 | 0.8 | 1.2 | 0.9 |
| **03-50** | pOIL103+pOIL197 | 1.4 | 1.8 | 1.3 |
| **03-51** | pOIL103+pOIL197 | 29.0 | 24.0 | 15.7 |
| **03-52** | pOIL103+pOIL197 | 30.2 | 14.3 | 16.4 |
| **03-53** | pOIL103+pOIL197 | 32.0 | 27.0 | 25.0 |
| **03-54** | pOIL103+pOIL197 | 41.0 | 14.7 | 17.4 |
| **03-55** | pOIL103+pOIL197 | 1.4 | 0.0 | 1.3 |
| **03-56** | pOIL103+pOIL197 | 0.0 | 1.5 | 1.3 |
| **03-57** | pOIL103+pOIL197 | 20.6 | 10.4 | 8.3 |
| **03-58** | pOIL103+pOIL197 | 17.9 | 4.2 | 15.3 |
| **03-59** | pOIL103+pOIL197 | 8.4 | 0.0 | 0.0 |
| **03-60** | pOIL103+pOIL197 | 54.0 | 23.6 | 25.6 |
| **03-61** | pOIL103+pOIL197 | 35.0 | 9.9 | 5.3 |
| **03-62** | pOIL103+pOIL197 | 2.6 | 3.1 | 4.4 |
| **03-63** | pOIL103+pOIL197 | 40.0 | 17.2 | 12.1 |

**Supplementary Table 3.** Transgene expression levels in propagated tillers of selected lines normalized to *S. bicolor* *actin.*

| **Line** | **Construct(s)** | **WRI1** | **DGAT2a** | **Oleosin-L** |
| --- | --- | --- | --- | --- |
| **WT 1** |  | 0.00 | 0.00 | 0.04 |
| **WT 2** |  | 0.00 | 0.00 | 0.01 |
| **01-2** | pOIL102 | 13.90 | 0.19 | 0.02 |
| **01-3** | pOIL102 | 5.47 | 0.07 | 0.00 |
| **01-4** | pOIL102 | 0.00 | 0.01 | 0.01 |
| **01-5** | pOIL102 | 0.00 | 0.05 | 0.00 |
| **01-6** | pOIL102 | 8.10 | 0.60 | 0.00 |
| **01-7** | pOIL102 | 3.53 | 0.14 | 0.00 |
| **01-8** | pOIL102 | 0.06 | 0.10 | 0.00 |
| **01-9** | pOIL102 | 0.01 | 0.11 | 0.02 |
| **197-8** | pOIL197 | 0.00 | 71.00 | 0.49 |
| **197-14** | pOIL197 | 0.01 | 56.00 | 12.00 |
| **197-19** | pOIL197 | 0.01 | 50.00 | 27.50 |
| **197-23** | pOIL197 | 0.00 | 88.00 | 2.26 |
| **02-1** | pOIL102+pOIL197 | 0.25 | 0.41 | 1.18 |
| **02-2** | pOIL102+pOIL197 | 2.40 | 14.80 | 0.55 |
| **02-4** | pOIL102+pOIL197 | 0.06 | 59.20 | 0.08 |
| **02-6** | pOIL102+pOIL197 | 6.50 | 146.00 | 7.50 |
| **02-10** | pOIL102+pOIL197 | 3.20 | 290.00 | 5.70 |
| **02-12** | pOIL102+pOIL197 | 0.24 | 21.00 | 4.22 |
| **02-13** | pOIL102+pOIL197 | 0.00 | 28.70 | 0.60 |
| **02-14** | pOIL102+pOIL197 | 2.10 | 17.00 | 0.57 |
| **02-16** | pOIL102+pOIL197 | 9.00 | 200.00 | 7.20 |
| **02-18** | pOIL102+pOIL197 | 0.01 | 9.40 | 0.88 |
| **02-19** | pOIL102+pOIL197 | 10.60 | 270.00 | 3.32 |
| **02-30** | pOIL102+pOIL197 | 10.80 | 101.00 | 0.61 |
| **02-31** | pOIL102+pOIL197 | 0.26 | 0.79 | 0.62 |
| **02-34** | pOIL102+pOIL197 | 0.09 | 200.00 | 12.60 |
| **02-38** | pOIL102+pOIL197 | 13.00 | 160.00 | 5.01 |
| **02-40** | pOIL102+pOIL197 | 0.50 | 0.08 | 0.78 |
| **02-41** | pOIL102+pOIL197 | 0.00 | 30.20 | 0.57 |
| **03-2** | pOIL103+pOIL197 | 24.00 | 44.00 | 1.00 |
| **03-5** | pOIL103+pOIL197 | 3.30 | 10.30 | 0.75 |
| **03-7** | pOIL103+pOIL197 | 64.00 | 0.02 | 1.47 |
| **03-8** | pOIL103+pOIL197 | 11.60 | 6.10 | 2.77 |
| **03-11** | pOIL103+pOIL197 | 106.00 | 132.00 | 7.70 |
| **03-17** | pOIL103+pOIL197 | 6.50 | 28.00 | 0.27 |
| **03-25** | pOIL103+pOIL197 | 21.00 | 25.00 | 8.90 |
| **03-26** | pOIL103+pOIL197 | 22.00 | 42.00 | 5.60 |
| **03-31** | pOIL103+pOIL197 | 137.00 | 290.00 | 3.83 |
| **03-36** | pOIL103+pOIL197 | 12.20 | 17.00 | 1.02 |
| **03-46** | pOIL103+pOIL197 | 16.50 | 103.00 | 1.82 |
| **03-48** | pOIL103+pOIL197 | 32.00 | 130.00 | 3.43 |
| **03-53** | pOIL103+pOIL197 | 9.00 | 70.00 | 38.00 |
| **03-55** | pOIL103+pOIL197 | 0.20 | 0.02 | 1.11 |

**
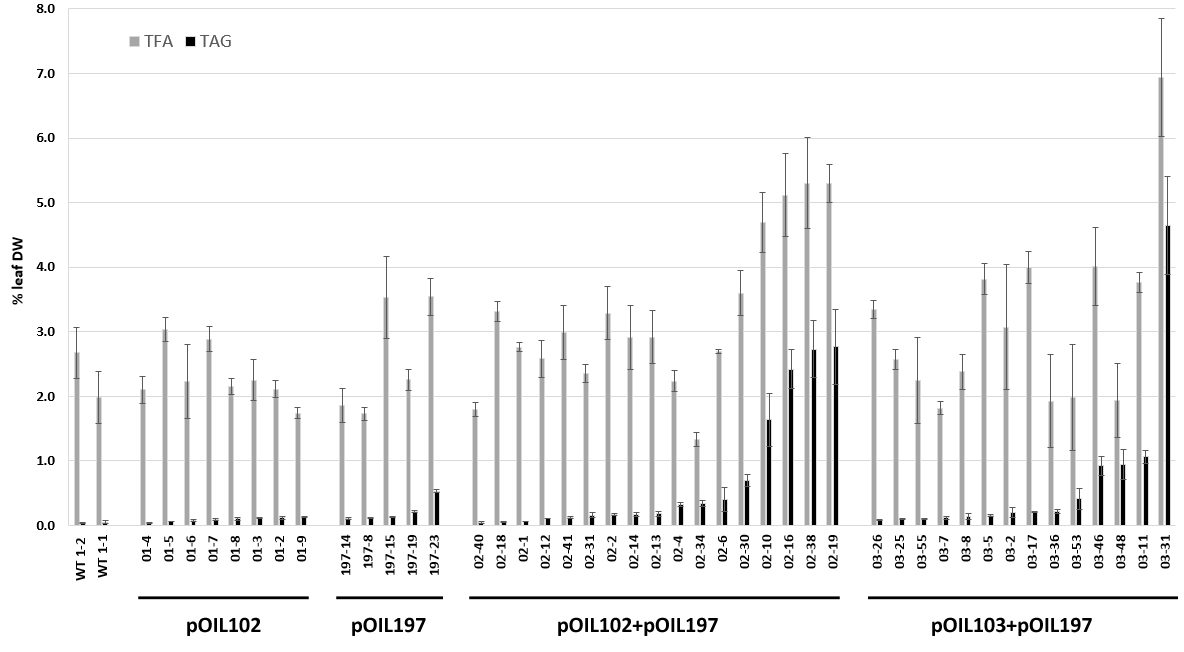
**

**Supplementary Figure 1.** Total lipids (TFA) and triacylglycerol (TAG) contents on a dry weight basis (DW) in leaves of selected transgenic *Sorghum bicolor* at boot leaf stage. Sorghum was transformed with pOIL102, pOIL197, pOIL102+pOIL197 or pOIL103+pOIL197. Selected primary transformants were re-established from side-tillers obtained from selected independent primary transformants. Means and standards deviations are based on triplicate leaves for each transgenic line.

**
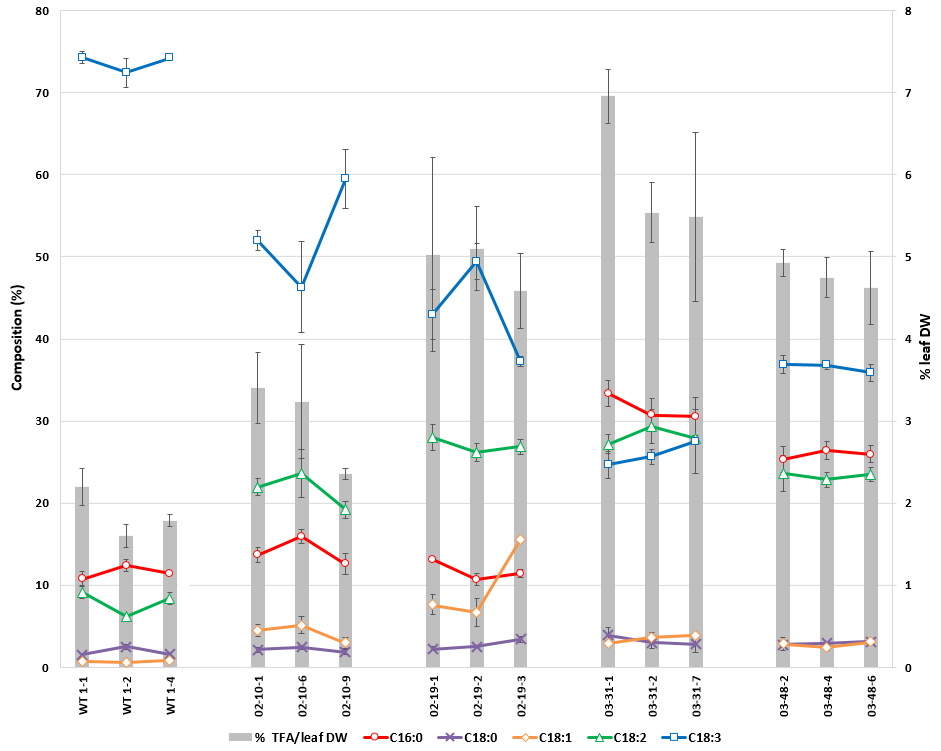
**

**Supplementary Figure 2.** Total lipid (TFA) composition and content on a dry weight basis (DW) in leaves of wild-type and transgenic *Sorghum bicolor* at boot leaf stage. Only major fatty acids are shown. Triplicate tillers were propagated from four selected independent primary transgenic events, transformed either with pOIL102+pOIL197 (‘02’ events) or pOIL103+pOIL197 (‘03’ events). Means and standards deviations are based on triplicate leaves from each propagated tiller.

**
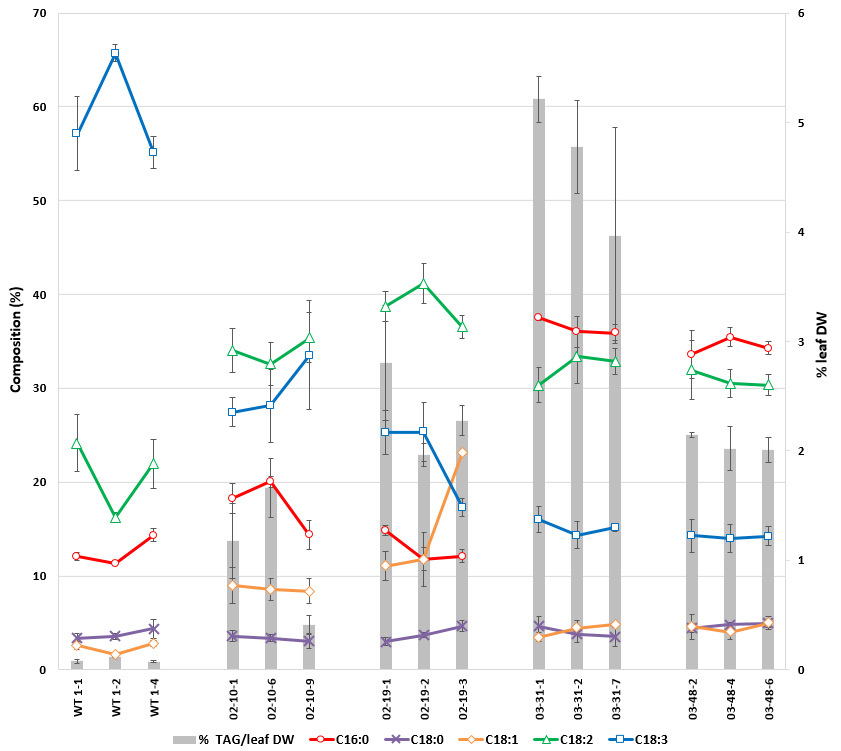
**

**Supplementary Figure 3.** Triacylglycerol (TAG) composition and content on a dry weight basis (DW) in leaves of wild-type and transgenic *Sorghum bicolor* at boot leaf stage. Only major fatty acids are shown. Triplicate tillers were propagated from four selected independent primary transgenic events, transformed either with pOIL102+pOIL197 (‘02’ events) or pOIL103+pOIL197 (‘03’ events). Means and standards deviations are based on triplicate leaves from each propagated tiller.

**
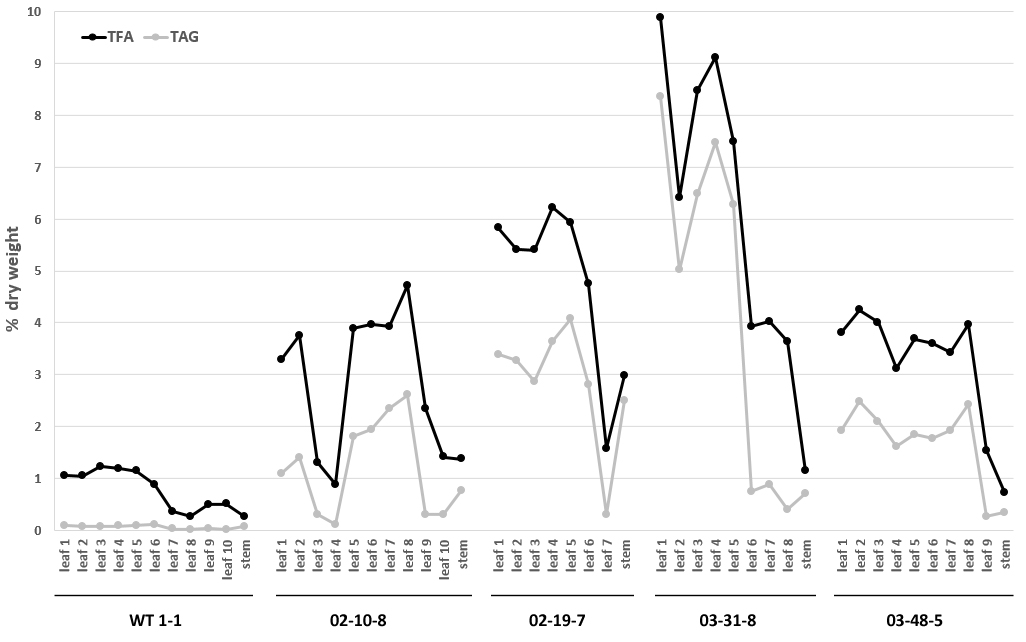
**

**Supplementary Figure 4.** Total lipid (TFA) and triacylglycerol (TAG) contents (% dry weight) in leaf and stem tissues of wild-type and transgenic *Sorghum bicolor* at seed setting stage. Transgenic ‘02’ and ‘03’ events were transformed with pOIL102+pOIL197 or pOIL103+pOIL197, respectively. Leaves from propagated tillers were numbered from top to bottom, starting from the flag leaf. Stem tissues were sampled from the bottom third part of the tiller. Values are based on single measurements.


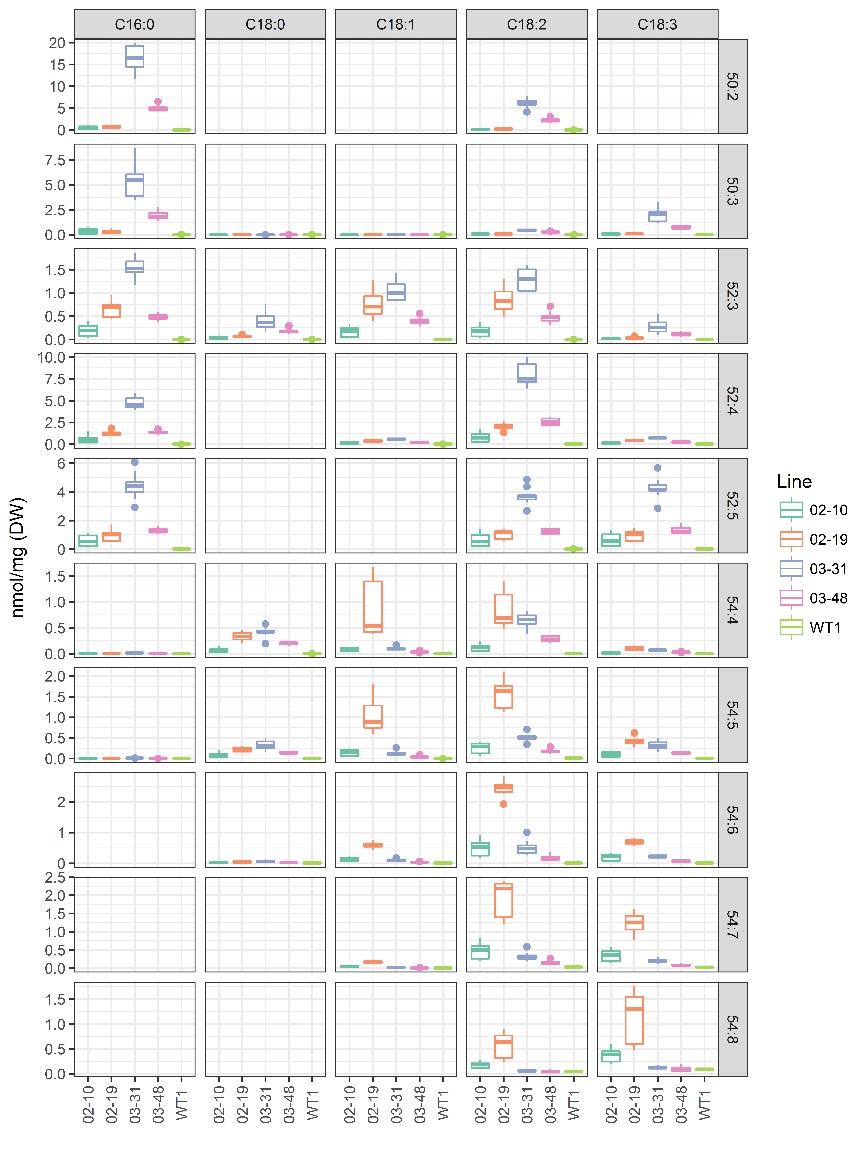


**Supplementary Figure 5.** Fatty acid composition of selected TAG species in leaves of wild-type and transgenic *Sorghum bicolor* at boot leaf stage. Triplicate tillers were propagated from four selected independent primary transgenic events, transformed either with pOIL102+pOIL197 (‘02’ events) or pOIL103+pOIL197 (‘03’ events). Individual TAG species are labelled based on the total number of carbon atoms and double bonds. Means and standards deviations are based on triplicate leaves from each propagated tiller.


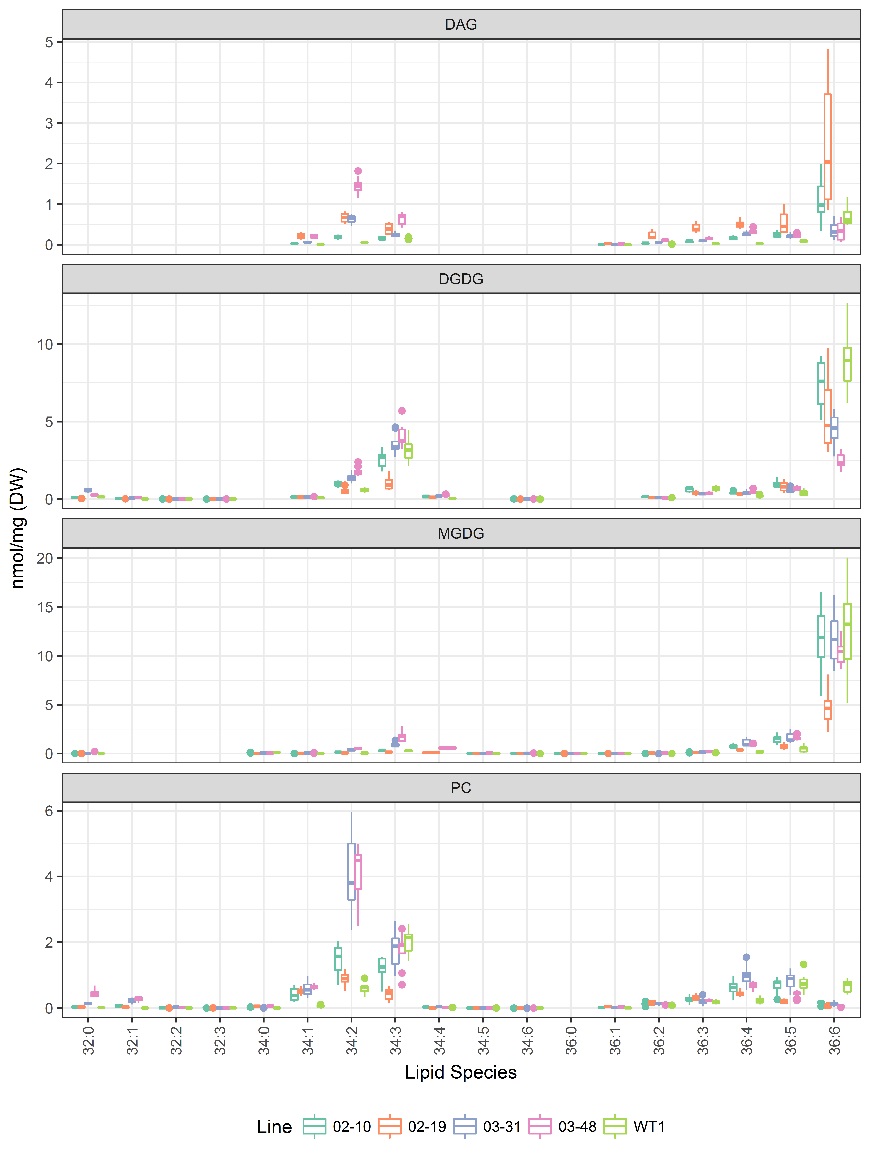


**Supplementary Figure 6.** Diacylglycerol (DAG), digalactosyldiacylglycerol (DGDG), monogalacosyldiacylglycerol (MGDG) and phosphatidylcholine (PC) molecular species (number of carbon atoms: number of double bonds) in wild-type and transgenic *Sorghum bicolor* leaves at boot leaf stage. Lines ‘02’ and ‘03’ were transformed with pOIL102+pOIL197 or pOIL103+pOIL197, respectively. Means and standards deviations are based on triplicate leaves from triplicate propagated tillers.

**
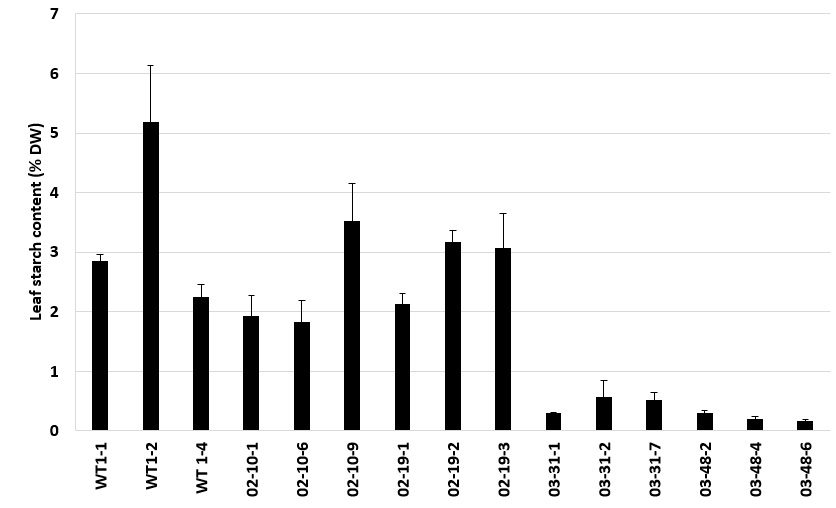
**

**Supplementary Figure 7.** Starch content (% dry weight) in leaves of wild-type and transgenic *Sorghum bicolor* at boot leaf stage. Triplicate tillers were propagated from four selected independent primary transgenic events, transformed either with pOIL102+pOIL197 (‘02’ events) or pOIL103+pOIL197 (‘03’ events). Means and standards deviations are based on triplicate leaves from each propagated tiller.

**
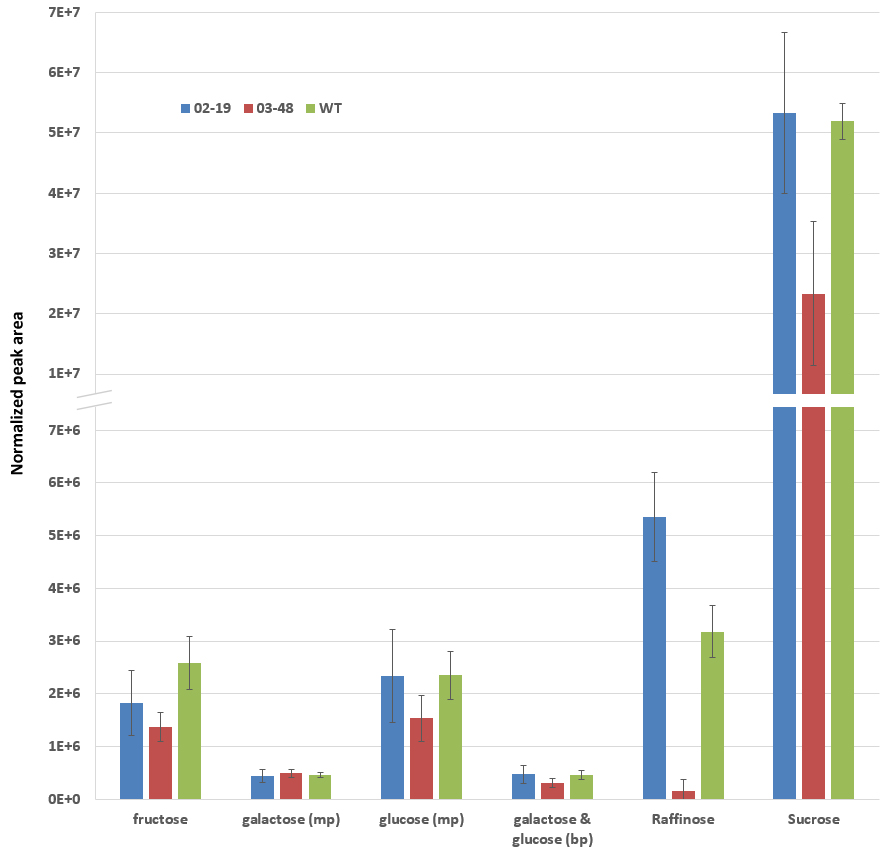
**

**Supplementary Figure 8.** Relative contents of different soluble sugars in leaves of wild-type and transgenic *Sorghum bicolor* at boot leaf stage. Transgenic lines 02-19 and 03-48 were transformed either with pOIL102+pOIL197 or pOIL103+pOIL197, respectively. Glucose and galactose were detected as two peaks (‘mp’, major product; ‘bp’, biproduct) with identical mass spectra. Means and standards deviations are based on triplicate leaves from 2-3 propagated tillers.

**
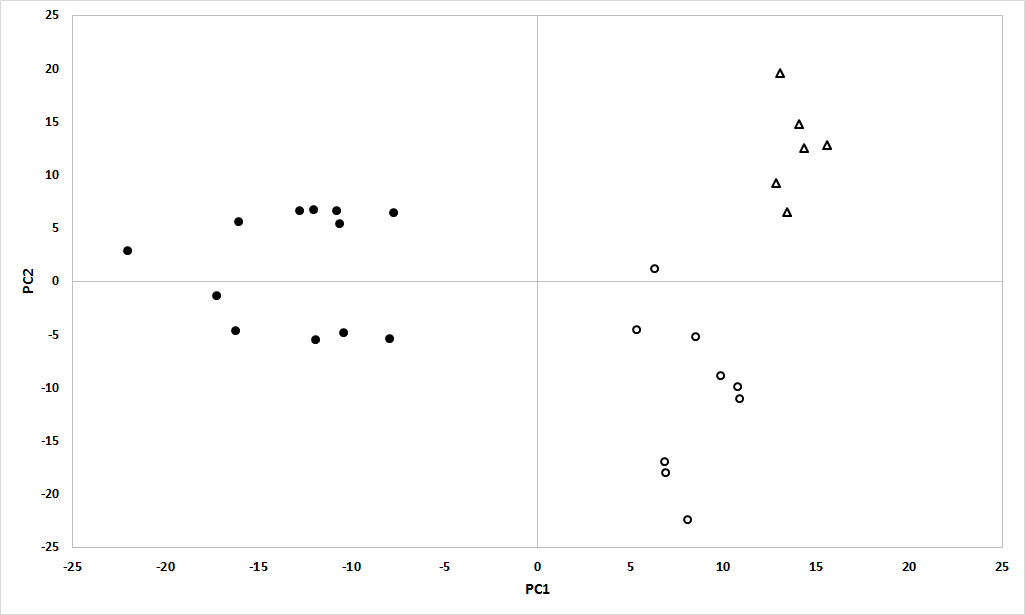
**

**Supplementary Figure 9.** Principle component analysis (PCA) of metabolites in wild-type and transgenic *Sorghum bicolor* leaves at boot leaf stage. Wild-type (open triangles), lines 02-19 (open circles) and 03-48 (closed circles) are clustered.

**
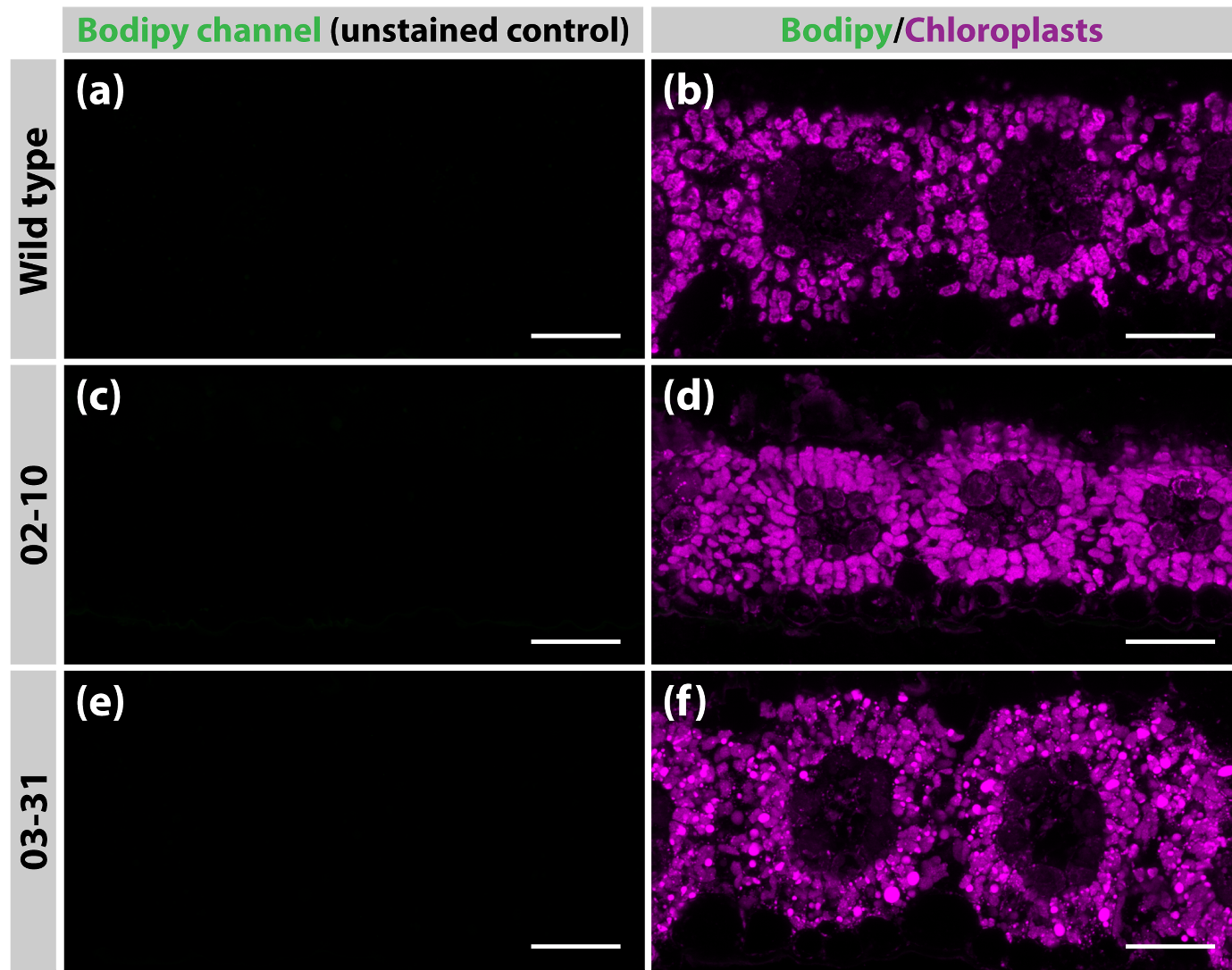
Supplementary Figure 10.** Confocal images of unstained fresh *Sorghum bicolor* leaf cross-sections of wild-type (a,b) or propagated tillers transformed with either pOIL102+pOIL197 (c,d) or pOIL103+pOIL197 (e-f), showing that there was no autofluorescence in the Bodipy channel (green, a,c,e). Chloroplast autofluorescence was used to highlight the mesophyll chloroplasts (magenta, b,d,f,h,i). Scale bars: 40µm.
